# Treatment Outcomes of Childhood Tuberculous Meningitis in a Real-World Retrospective Cohort, Bandung, Indonesia

**DOI:** 10.3201/eid2803.212230

**Published:** 2022-03

**Authors:** Heda M. Nataprawira, Fajri Gafar, Nelly A. Risan, Diah A. Wulandari, Sri Sudarwati, Ben J. Marais, Jasper Stevens, Jan-Willem C. Alffenaar, Rovina Ruslami

**Affiliations:** Hasan Sadikin Hospital, Bandung, Indonesia (H.M. Nataprawira, N.A. Risan, D.A. Wulandari, S. Sudarwati);; Universitas Padjadjaran, Bandung, Indonesia (H.M. Nataprawira, N.A. Risan, D.A. Wulandari, S. Sudarwati, R. Ruslami);; University of Groningen, Groningen, the Netherlands (F. Gafar, J. Stevens);; Children’s Hospital at Westmead, Sydney, New South Wales, Australia (B.J. Marais);; University of Sydney, Sydney (B.J. Marais, J.-W.C. Alffenaar);; Westmead Hospital, Sydney (J.-W.C. Alffenaar)

**Keywords:** tuberculosis, tuberculous meningitis, tuberculosis and other mycobacteria, children, treatment outcome, mortality, morbidity, neurologic sequelae, bacteria, Indonesia

## Abstract

We retrospectively evaluated clinical features and outcomes in children treated for tuberculous meningitis (TBM) at Hasan Sadikin Hospital, Bandung, Indonesia, during 2011–2020. Among 283 patients, 153 (54.1%) were <5 years of age, and 226 (79.9%) had stage II or III TBM. Predictors of in-hospital death (n = 44 [15.5%]) were stage III TBM, hydrocephalus, male sex, low-income parents, seizures at admission, and lack of bacillus Calmette-Guérin vaccination. Predictors of postdischarge death (n = 18 [6.4%]) were hydrocephalus, tuberculoma, and lack of bacillus Calmette-Guérin vaccination. At treatment completion, 91 (32.1%) patients were documented to have survived, of whom 33 (36.3%) had severe neurologic sequelae and 118 (41.7%) had unknown outcomes. Predictors of severe neurologic sequelae were baseline temperature >38°C, stage III TBM, and baseline motor deficit. Despite treatment, childhood TBM in Indonesia causes substantial neurologic sequelae and death, highlighting the importance of improved early diagnosis, better tuberculosis prevention, and optimized TBM management strategies.

Tuberculosis (TB) is a major global health problem, and an estimated 1.2 million new pediatric cases and 230,000 deaths occurred in children <15 years of age in 2019 (*1*). Tuberculous meningitis (TBM) is the most severe manifestation of TB, leading to high rates of childhood TBM mortality, at an average of 19%, and neurodisability in >50% of survivors, even when treatment is provided (*2*). After infection with *Mycobacterium tuberculosis*, children <2 years of age are at the highest risk for progression to miliary TB and TBM, most likely because of their immature immune systems (*3*). Childhood and adolescent TB has historically been neglected (*4*,*5*); however, recently this condition has begun to gain priority as a focus of global collaborative efforts toward ending TB in children and adolescents (*6*).

The most important predictors of favorable outcome in childhood TBM are early diagnosis and immediate initiation of treatment (*2*). However, incomplete understanding of the pathogenesis, nonspecific symptoms, suboptimal performance of diagnostic tests, and the paucibacillary nature of the disease often result in a lengthy process of obtaining a definite diagnosis (*7*–*9*). Moreover, antimicrobial therapy as currently recommended by the World Health Organization (WHO) for the management of childhood TBM remains suboptimal (*9*,*10*) and most likely contributes to poor outcomes. Summary estimates of neurologic sequelae and death associated with childhood TBM have been described in a meta-analysis, but predictors of these poor outcomes other than diagnosis in the most advanced disease stage were reported to have high heterogeneities across studies (*2*). Data on clinical features and treatment outcomes of childhood TBM from large cohorts of children outside of South Africa are limited (*11*–*13*). In settings in Indonesia, a few small studies have reported clinical outcomes of childhood TBM (*14*–*16*), but none have explored factors associated with the outcomes. This characterization is clinically relevant, enabling early and targeted interventions to optimize care in this vulnerable population. In this context, our study aimed to assess clinical features of childhood TBM and to evaluate factors associated with poor outcomes, including in-hospital death, postdischarge death, and neurologic sequelae.

## Methods

### Patients and Setting

This real-world retrospective cohort study consecutively included children <15 years of age treated for TBM at the Department of Child Health of Hasan Sadikin Hospital, a national tertiary teaching hospital in Bandung, Indonesia, during January 2011–December 2020. The study was approved by the Independent Ethics Committee of Hasan Sadikin Hospital (approval no. LB.02.01/X.6.5/91/2021). Because of the retrospective nature of the study design, the Ethics Committee waived the need for written informed consent.

### Diagnosis

We established TBM diagnosis on the basis of clinical, laboratory, and radiologic findings (*17*), combining medical history, physical and clinical examinations, tuberculin skin test, chest radiography, cerebrospinal fluid (CSF) analysis, and neuroimaging by using computed tomography (CT) scan. We performed microbiologic examination of CSF and non-CSF samples, including smear microscopy for acid-fast bacilli (AFB), culture for *M. tuberculosis*, and Xpert MTB/RIF assay, depending on sample availability. We assessed diagnostic certainty of definite, probable, or possible TBM by using uniform case definition criteria for TBM research (*18*) (Appendix Table 1). We presumed that patients had drug-susceptible TBM unless drug resistance was proven in Xpert MTB/RIF or drug-susceptibility testing. We excluded TBM patients with drug-resistant TB from the study.

**Table 1:** Demographic and clinical characteristics at admission of children with TBM treated at Hasan Sadikin Hospital, Bandung, Indonesia, 2011–2020*

Characteristic	Total patients			<5 y			5–14 y	
	No.†	Value		No.†	Value		No.†	Value
Age, y , median (IQR)	283	4.0 (1.0; 10.0)		153	1.0 (0.7–2.4)		130	10.2 (8.0–12.2)
Sex								
M	283	150 (53.0)		153	74 (48.4)		130	76 (58.5)
F	283	133 (47.0)		153	79 (51.6)		130	54 (41.5)
Nutritional status‡								
WFAZ, median (IQR)	227	−2.2 (−3.0 to −1.0)		153	−1.9 (−2.9 to −0.7)		74	−2.5 (−3.2 to −1.7)
HFAZ, median (IQR)	283	−1.6 (−2.6 to −0.3)		153	−1.6 (−2.8 to −0.7)		130	−1.6 (−2.5 to −0.4)
BFAZ, median (IQR)	283	−2.1 (−3.2 to −0.4)		153	−1.7 (−2.8 to −0.2)		130	−2.6 (−3.7 to −0.5)
Moderately malnourished	283	74 (26.1)		153	44 (28.8)		130	30 (23.1)
Severely malnourished	283	109 (38.5)		153	47 (30.7)		130	62 (47.7)
Known BCG vaccination	283	223 (78.8)		153	120 (78.4)		130	103 (79.2)
Known TB contact history	283	73 (25.8)		153	36 (23.5)		130	37 (28.5)
Known HIV co-infection	283	4 (1.4)		153	0 (0.0)		130	4 (3.1)
Baseline temperature, °C, median (IQR)	282	37.0 (36.8–37.9)		153	37.2 (36.9–38.0)		129	37.0 (36.8–37.8)
Symptoms duration, d, median (IQR)§	269	8 (7–11)		145	8 (7–10)		124	9 (7–12)
Symptoms								
Fever	283	250 (88.3)		153	136 (88.9)		130	114 (87.7)
Severe headache	278	61 (21.9)		150	13 (8.7)		128	48 (37.5)
Muscle weakness	278	73 (26.3)		151	40 (26.5)		127	33 (26.0)
Altered consciousness	283	211 (74.6)		153	111 (72.5)		130	100 (76.9)
Seizures	282	155 (55.0)		153	84 (54.9)		129	71 (55.0)
Persistent cough	282	95 (33.7)		152	53 (34.9)		130	42 (32.3)
Poor weight gain or weight loss	279	105 (37.6)		151	51 (33.3)		128	54 (41.5)
Motor function								
Hemiparesis	263	51 (19.4)		142	27 (19.0)		121	24 (19.8)
Quadriparesis	263	95 (36.1)		142	59 (41.5)		121	36 (29.8)
Cranial nerve palsy	277	48 (17.3)		149	31 (20.8)		128	17 (13.3)
Signs of upper motor neuron lesion	264	188 (71.2)		143	93 (65.0)		121	95 (78.5
Signs of raised intracranial pressure	283	47 (16.6)		153	29 (19.0)		130	18 (13.8)
TBM category¶								
Definite	283	51 (18.0)		153	26 (17.0)		130	25 (19.2)
Probable	283	178 (62.9)		153	101 (66.0)		130	77 (59.2)
Possible	283	54 (19.1)		153	26 (17.0)		130	28 (21.5)
GCS, median (IQR)	283	12 (10–14)		153	12 (10–15)		130	12 (10–14)
TBM stage#								
Stage I	283	57 (20.1)		153	35 (22.9)		130	22 (16.9)
Stage II	283	131 (46.3)		153	60 (39.2)		130	71 (54.6)
Stage III	283	95 (33.6)		153	58 (37.9)		130	37 (28.5)

*Values are no. (%) or median (IQR) except as indicated. BCG, bacillus Calmette-Guérin; BFAZ, body mass index-for-age Z-score; GCS, Glasgow Coma Scale; HFAZ, height-for-age Z-score; IQR, interquartile rage; TB, tuberculosis; TBM, tuberculous meningitis; WFAZ, weight-for-age Z-score. †Number of total patients for whom data were available (denominator). ‡In children <5 years of age, moderate malnutrition was defined as WFAZ or HFAZ ≥–3 but <–2 standard deviation (SD), and severe malnutrition as WFAZ or HFAZ <–3 SD. In children aged 5–14 y, moderate malnutrition was defined as HFAZ or BFAZ ≥–3 but <–2 SD, and severe malnutrition as HFAZ or BFAZ <–3 SD. §Duration of symptoms before admission. ¶Diagnostic certainty was categorized as definite TBM (microbiologically proven from CSF examination), probable TBM (diagnostic score of ≥10 when neuroimaging was unavailable or ≥12 when neuroimaging was available), and possible TBM (diagnostic score of 6–9 when neuroimaging was unavailable or 6–11 when neuroimaging was available) (*18*). #TBM staging was classified according to the modified British Medical Research Council grading system as stage I (GCS of 15 with no focal neurologic signs), stage II (GCS 11–14 or 15 with focal neurologic signs), or stage III (GCS ≤10) (*20*).

### Treatment

We based treatment regimens on the 2010 WHO guidelines in accordance with the Indonesian Paediatric Society guidelines for TBM treatment in children, consisting of daily isoniazid at 10 mg/kg (range 7–15 mg/kg), rifampin at 15 mg/kg (range 10–20 mg/kg), pyrazinamide at 35 mg/kg (range 30–40 mg/kg), and ethambutol at 20 mg/kg (range 15–25 mg/kg) for a 2-month intensive phase, followed by a 10-month continuation phase with isoniazid and rifampin at the same doses (*17*,*19*). We administered all anti-TB drugs orally as fixed-dose combination or single-drug formulation tablets, where available. Patients received facility-based directly observed therapy (DOT) during hospitalization. After discharge, patients received home-based DOT under the supervision of parents or other family members. Most patients received adjunctive oral prednisone (2–4 mg/kg/d) for the first 4–8 weeks, tapered according to the national guidelines (*17*). We treated patients with increased intracranial pressure with hypertonic saline or mannitol 20% (0.5–1 g/kg) every 8 hours. We performed ventriculoperitoneal shunt or extraventricular drain placements in patients with obstructive hydrocephalus, at the discretion of the neurosurgical team.

### Data Collection

We collected individual patient data from hospital registry in a predefined form and appropriately deidentified the data before analysis. These data were demographic information (age, sex, parents’ education and income, area of living, and length of hospital admission); medical history (HIV infection, bacillus Calmette-Guérin [BCG] vaccination, and TB contact history); clinical characteristics (symptoms of TBM, vital signs, nutritional status, physical and neurologic examinations, tuberculin skin test, Glasgow coma scale [GCS], and TBM staging); laboratory findings (CSF analysis, AFB microscopy, mycobacterial culture, and Xpert MTB/RIF test); radiographic findings (chest radiograph and neuroimaging); and other supporting data (corticosteroid therapy and in-hospital complications).

### Definitions

We developed operational definitions for all variables (Appendix Table 2). We defined definite TBM as microbiologic confirmation of CSF and probable TBM as a total diagnostic score of >12 when neuroimaging was available or ≥10 when neuroimaging was unavailable. We defined possible TBM as a total score of 6–11 when neuroimaging was available or 6–9 when neuroimaging was unavailable (*18*). We classified TBM staging according to the modified British Medical Research Council grading system (*20*), as follows: stage I, GCS 15 without focal neurologic deficit; stage II, GCS 11–14, or 15 with focal neurologic deficit; and stage III, GCS <10. Patients with known BCG vaccination included those who had a documented vaccination history at hospital admission or had a BCG scar in the deltoid region of the upper arm. Motor deficits included hemiparesis, quadriparesis, and diplegia. Other neurologic deficits were signs of upper motor neuron lesion and cranial nerve palsies. We performed motor, hearing, visual, and neurodevelopmental function assessments at treatment completion as indicated by the attending physicians (Appendix).

**Table 2:** Laboratory and radiographic findings at admission of children with tuberculous meningitis treated at Hasan Sadikin Hospital, Bandung, Indonesia, 2011–2020*

Characteristic	Total patients			Age <5 y			Age 5–14 y	
	No.†	Value		No.†	Value		No.†	Value
CSF analysis, median (IQR)								
Leukocytes, cells/µL	276	44 (11–109)		149	56 (14–117)		127	40 (8–95)
Protein, mg/dL	276	107 (60–239)		151	103 (68–234)		125	120 (46–248)
MN, %	275	83 (60–96)		151	81 (60–95)		124	86 (64–98)
PMN, %	275	15 (4–37)		151	18 (5–40)		124	12 (0.2–36)
Glucose, mg/dL	269	47 (25–66)		150	42 (20–67)		119	52 (34–66)
CSF-to-plasma glucose ratio, median (IQR)	241	0.4 (0.2–0.6)		140	0.4 (0.2–0.6)		101	0.5 (0.3–0.6)
Cerebral imaging‡								
Hydrocephalus	250	103 (41.2)		136	64 (47.1)		114	39 (34.2)
Basal meningeal enhancement	250	131 (52.4)		136	74 (54.4)		114	57 (50.0)
Infarct	250	25 (10.0)		136	12 (8.8)		114	13 (11.4)
Tuberculoma	250	31 (12.4)		136	17 (12.5)		114	14 (12.3)
Chest radiography								
Miliary TB	281	19 (6.8)		152	10 (6.6)		129	9 (7.0)
Other signs of active TB	281	128 (45.6)		152	66 (43.4)		129	62 (48.1)
TST positive§	283	64 (22.6)		153	37 (24.2)		130	27 (20.8)
*M. tuberculosis* cultured from any source¶	267	26 (9.7)		147	15 (10.2)		120	11 (9.2)
AFB smear microscopy								
Positive from CSF	272	6 (2.2)		149	4 (2.7)		123	2 (1.6)
Positive from any non-CSF sample#	282	49 (17.4)		152	23 (15.1)		130	26 (20.0)
Xpert MTB/RIF testing**								
Positive from CSF	140	48 (34.3)		77	24 (31.2)		63	24 (38.1)
Positive from gastric lavage	212	71 (33.5)		120	43 (35.8)		92	28 (30.4)
Positive from sputum	12	5 (41.7)		2	0		10	5 (50.0)

*Values are no. (%) or median (IQR) except as indicated. AFB, acid-fast bacilli; CSF, cerebrospinal fluid; IQR, interquartile rage; MN, mononuclear cells; PMN, polymorphonuclear cells; TB, tuberculosis; TST, tuberculin skin test. †Number of total patients for whom data were available (denominator). ‡Cerebral imaging results were obtained mostly from noncontrast brain computed tomography scan, or from magnetic resonance imaging, where available. §The median size of induration (minimum–maximum range) in patients with a positive TST result was 12 (10–30) mm and in patients with a negative TST result was 0 (0–8) mm. ¶Culture of *M. tuberculosis* from CSF is rarely performed in our setting, mostly because of the limited CSF volume available from lumbar puncture. From our experience, most of the non-CSF specimens were obtained from gastric lavage, and some specimens were obtained from sputum, but our data could not further specify the type of specimens used. Mycobacterial culture were mostly performed on solid media; the use of liquid culture media (MGIT, BACTEC) has only begun in recent years. #We could not further specify the types of non-CSF specimens used for AFB smear microscopy. **Data on Xpert MTB/RIF testing results have only been available since 2013.

### Outcomes

Outcomes of hospitalization were recovery (with or without disability), nonrecovery (persistent vegetative state and discharge against medical advice), and death. After 12 months of treatment, we reported the following outcomes: treatment completion, death, and lost to follow-up (LTFU; i.e., patients who stopped treatment for >2 consecutive months). “Not evaluated” or “unknown treatment outcome” categories were patients who were transferred back to regional public hospitals or community health clinics for follow-up after discharge. We defined survival as being alive at treatment completion and neurologic sequelae as any motor, hearing, visual, or neurodevelopmental impairment that appeared during the illness and persisted through treatment completion.

### Data Analysis

We evaluated associations of patient characteristics with poor outcomes. First, we compared patients who died during hospitalization (in-hospital death) with those who had recovered at the time of discharge; this definition excluded persistent vegetative state and discharge against medical advice. Second, we compared patients who died after discharge (postdischarge death) with those who completed treatment, regardless of their sequelae status; this definition excluded LTFU and unknown outcomes. Third, we compared survivors with neurologic sequelae with those without sequelae; this definition excluded death, LTFU, and unknown outcomes.

We used Cox proportional-hazards regression analysis to assess predictors of in-hospital death. We calculated time to death on the basis of length of stay by subtracting day of admission from day of death. Most patients were discharged within 2 months of hospitalization; in this case, we assumed that recovering patients (with or without disability) discharged before 2 months were alive until the end of 2 months, and thus we censored these patients in the Cox regression analysis. Because the time to death after discharge was not recorded, we assessed associated factors with postdischarge death and neurologic sequelae by using logistic regression analysis. We adjusted our multivariate models for age, sex, and TBM staging, and completed the models with variables showing a trend toward association in univariate analysis. We selected these variables by using backward deletion, and the final models retained all additional variables with a p value <0.1. For logistic regression analysis, we evaluated the goodness-of-fit of the final models by using Hosmer-Lemeshow test and performance by the area under the receiver operating characteristic curve. For Cox regression analysis, we checked proportional hazards assumption using Kaplan-Meier curve before fitting the model, and using log-minus-log survival curve after fitting the model. We used adjusted hazard ratios (aHRs) for Cox regression models and adjusted odds ratios (aORs) for logistic regression models, as well as 95% CIs, to estimate the association between explanatory variables and outcomes. We defined statistical significance as p<0.05. We performed all analyses by using IBM SPSS Statistics 26.0 (https://www.ibm.com).

## Results

### Clinical Characteristics

During the study period (2011–2020), 286 children with TBM were treated at Hasan Sadikin Hospital; 3 patients with rifampin-resistant TB were excluded. No patients had concurrent bacterial meningitis. Among 283 included patients, 150 (53.0%) were boys, 153 (54.1%) were <5 years of age, 183 (64.7%) were malnourished, 226 (79.9%) had stage II or III TBM, and 51 (18.0%) had definite TBM. At admission, most patients had history of fever (88.3%), decreased consciousness (74.6%), and seizures (55.0%); the next most common signs and symptoms were weight loss (37.6%), persistent cough (33.7%), muscle weakness (26.3%), and severe headache (21.9%). These signs and symptoms had existed for >5 days before admission in 87.0% of patients (Table 1). We stratified manifestations by disease staging (Appendix Table 3). 

In CSF analysis, most patients had pleocytosis (>10 cells/µL, 76.8%), and lymphocytic predominance (>50%, 81.8%), followed by a low CSF-to-plasma glucose ratio (<0.5, 54.8%), elevated protein level (>100 mg/dL, 51.8%), and hypoglycorrhachia (<40 mg/dL, 41.6%). *M. tuberculosis* susceptible to rifampin was identified by Xpert MTB/RIF assay in 48 (34.3%) of 140 CSF samples and in 76 (33.9%) of 224 non-CSF samples. In neuroimaging, most patients had basal meningeal enhancement (52.4%), followed by hydrocephalus (41.2%), tuberculoma (12.4%), and infarct (10.0%) (Table 2). Among 103 patients with hydrocephalus, 45 (43%) received neurosurgical intervention: 44 (97.8%) ventriculoperitoneal shunt and 1 (2.2%) extraventricular drain. 

For in-hospital complications, 106 (37.5%) of the 283 patients had motor disorders, 37 (13.1%) had neurodevelopmental delay, 19 (6.7%) had epileptic seizures, 17 (6.0%) had visual impairment, 12 (4.2%) had hearing impairment, and 27 (9.5%) had anti-TB drug-induced hepatotoxicity. Adjunctive oral corticosteroid was administered to 262 (92.6%) of patients. In addition, 1 of the patients (a 6-month-old boy with stage II TBM) had severe acute respiratory syndrome coronavirus 2 coinfection (Appendix).

### In-Hospital Death

Upon discharge, 231 (81.6%) of 283 patients had recovered (with or without disability), 3 (1.1%) had a persistent vegetative state, and 5 (1.8%) were discharged against medical advice. The remaining 44 (15.5%) died; median time to death was 7 days (interquartile range 3–13 days) after admission (Table 3). 

**Table 3:** Hospitalization and end of treatment outcome, stratified by disease staging, in children with tuberculous meningitis treated at Hasan Sadikin Hospital, Bandung, Indonesia, 2011–2020*

Variable	Total	Stage I†	Stage II†	Stage III†
Outcome of hospitalization‡				
Cases, no.	283	57	131	95
Recovered	231 (81.6)	54 (94.7)	111 (84.7)	66 (69.5)
Not recovered	8 (2.8)	1 (1.8)	5 (3.8)	2 (2.1)
Died	44 (15.5)	2 (3.5)§	15 (11.5)	27 (28.4)
Length of hospital stay, d, median (IQR)	10 (7–17)	9 (7–14)	10 (7–14)	15 (8–25)
Time to death, d, median (IQR)	7 (3–13)	(4–14)¶	6 (2–8)	8 (3–16)
Outcome at treatment completion‡#				
Cases, no.	272	56	122	94
Completed treatment	91 (33.5)	22 (39.3)	45 (36.9)	24 (25.5)
Without neurologic sequelae**	58 (63.7)	17 (77.3)	31 (68.9)	10 (41.7)
With neurologic sequelae**	33 (36.3)	5 (22.7)	14 (31.1)	14 (58.3)
Died	62 (22.8)	2 (3.6)	22 (18.0)	38 (40.4)
Died after hospital discharge	18 (6.6)	0 (0.0)	7 (5.7)	11 (11.7)
Lost to follow-up	1 (0.4)	0 (0.0)	1 (0.8)	0 (0.0)
Unknown treatment outcome	118 (43.4)	32 (57.1)	54 (44.3)	32 (34.0)

*Values are no. (%) except as indicated. IQR, interquartile rage. †Stage I was defined as Glasgow Coma Scale (GCS) of 15 with no focal neurologic signs, stage II as GCS of 11–14 or 15 with focal neurologic signs, and stage III as GCS ≤10 (*20*). ‡On hospital discharge, recovering patients were those who had clinical improvement (with or without disability), whereas non-recovering patients were those who had persistent vegetative state or discharged against medical advice. Treatment completion included patients who completed 12 mo of TBM therapy. Lost to follow-up included patients who stopped treatment for two consecutive months or more. Unknown treatment outcome included patients who were transferred back to regional public hospitals or community health clinics for follow-up after discharge. Neurologic sequelae were defined as any motor, hearing, visual, or neurodevelopmental impairment that appeared during the illness and persisted through treatment completion. §The causes of death in two patients with stage I TBM were hospital acquired pneumonia + thalassemia major (n = 1), and intracranial metastases of Burkitt lymphoma + increased intracranial pressure (n = 1). ¶Minimum–maximum range. #Excluding 11 patients who were still in ongoing treatment. **Percentages were calculated only in patients who completed 12 mo of treatment.

We performed univariate (Appendix Table 4) and multivariate (Table 4) analyses of risk for in-hospital death. In multivariate analysis, factors associated with increased risk were stage III TBM (aHR 5.96 [95% CI 1.39–25.58]), hydrocephalus (aHR 2.32 [95% CI 1.13–4.79]), male sex (aHR 2.10 [95% CI 1.09–4.05]), low-income parents (aHR 2.59 [95% CI 1.06–6.31]), seizures on admission (aHR 1.96 [95% CI 1.01–3.82]), and unknown BCG vaccination (aHR 1.97 [95% CI 1.03–3.76]). Among children <5 years of age, known history of TB contact was associated with an increased risk for in-hospital death (aHR 2.42 [95% CI 1.06–5.50]), adjusted for age, sex, and TBM staging. We charted Kaplan-Meier curves for several risk groups for in-hospital death (Figure).

**Table 4:** Multivariate Cox proportional-hazards regression model for factors associated with in-hospital death in children treated for TBM at Hasan Sadikin Hospital, Bandung, Indonesia, 2011–2020*

Variable	Died†‡	Alive†	Crude HR (95% CI)	p value	aHR (95% CI)	p value
No. cases	44	231				
Age, y						
<2	13 (29.5)	78 (33.8)	0.78 (0.37–1.67)	0.527	0.78 (0.36–1.68)	0.522
2–4	11 (25.0)	47 (20.3)	1.04 (0.47–2.29)	0.992	0.93 (0.41–2.12)	0.867
5–9	6 (13.6)	43 (18.6)	0.65 (0.25–1.70)	0.384	0.41 (0.15–1.11)	0.079
10–14	14 (31.8)	63 (27.3)	Referent		Referent	
Sex						
M	29 (65.9)	118 (51.1)	1.72 (0.92–3.20)	0.089	2.10 (1.09–4.05)	0.027
F	15 (34.1)	113 (48.9)	Referent		Referent	
TBM stage§,¶						
Stage I	2 (4.5)	54 (23.4)	Referent		Referent	
Stage II	15 (34.1)	111 (48.1)	3.53 (0.81–15.44)	0.094	2.57 (0.58–11.41)	0.214
Stage III	27 (61.4)	66 (28.6)	9.16 (2.18–38.51)	0.003	5.96 (1.39–25.58)	0.016
Parents’ monthly income#						
USD ≤140	33 (75.0)	136 (58.9)	2.79 (1.17–6.67)	0.021	2.59 (1.06–6.31)	0.036
USD >140	6 (13.6)	74 (32.0)	Referent		Referent	
Unknown	5 (11.4)	21 (9.1)	2.73 (0.83–8.95)	0.097	2.04 (0.59–7.02)	0.261
Known BCG vaccination						
No	15 (34.1)	44 (19.0)	2.01 (1.08–3.76)	0.028	1.97 (1.03–3.76)	0.040
Yes	29 (65.9)	187 (81.0)	Referent		Referent	
Hydrocephalus on CT¶						
No	12 (27.3)	133 (57.6)	Referent		Referent	
Yes**	22 (50.0)	76 (32.9)	3.00 (1.48–6.05)	0.002	2.32 (1.13–4.79)	0.022
Unknown	10 (22.7)	22 (9.5)	4.38 (1.89–10.13)	0.001	4.21 (1.77–10.01)	0.001
Seizures on admission¶						
No	13 (29.5)	112 (49.5)	Referent		Referent	
Yes	31 (70.5)	119 (51.5)	2.09 (1.09–3.99)	0.026	1.96 (1.01–3.82)	0.048

*Values are no. (%) except as indicated. aHR, adjusted hazard ratio; BCG, bacillus Calmette-Guérin; CT, computed tomography; GCS, Glasgow Coma Scale; IDR, Indonesian Rupiah; TBM, tuberculous meningitis. †Including patients who died or had recovered (with or without disability) on hospital discharge, and excluding patients who had persistent vegetative state or discharged against medical advice. ‡Signs of upper motor neuron lesion was associated with an increased risk of in-hospital death in univariate analysis, but did not remain significant in multivariate analysis. Signs of raised intracranial pressure with hydrocephalus as well as GCS score with TBM stage had the likelihood of collinearity; therefore, only hydrocephalus and TBM staging were included in the final multivariate model. For HIV coinfection, although it was significantly associated with in-hospital death in univariate analysis, we did not include this variable in multivariate analysis due to the selective HIV testing and a very low number of patients with HIV positive (n = 4). §Stage I TBM was defined as GCS of 15 with no focal neurologic signs, stage II TBM as GCS of 11–14 or 15 with focal neurologic signs, and stage III TBM as GCS ≤10 (*20*). ¶TBM staging might interact with hydrocephalus and seizures on admission; however, due to the low number of patients with stage I TBM who died during hospitalization (n = 2), these potential interactions could not be assessed in the Cox regression model. #Parents’ monthly income was estimated based on the current provincial minimum wage for West Java (IDR 1.810.350,00, rounded up to IDR 2.000.000,00, equal to approximately USD 140). **In-hospital death among children with hydrocephalus was not significantly different between those who received neurosurgical intervention and who did not receive neurosurgical intervention (p = 0.604).

**Figure:**
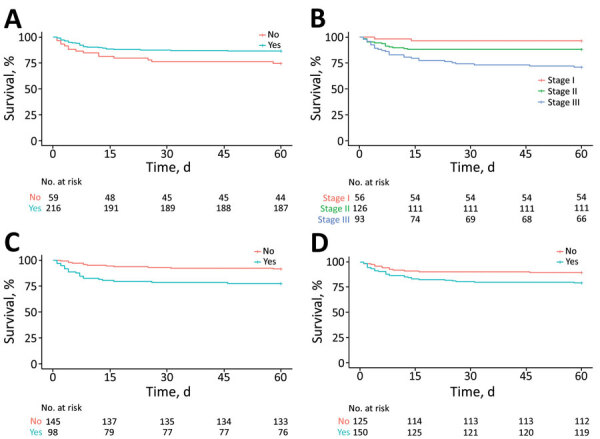
Survival curves for in-hospital death in children treated for tuberculous meningitis at Hasan Sadikin Hospital, Bandung, Indonesia, 2011–2020. A) Known bacillus Calmette-Guérin (BCG) vaccination status (yes/no); B) tuberculous meningitis stage (I–III); C) radiographic evidence of hydrocephalus (yes/no); D) presence of seizures at hospital admission (yes/no).

### Postdischarge Death

After the 12-month follow-up, 272 (96.1%) of 283 patients were evaluated for treatment outcomes, and 11 (3.9%) in ongoing treatment who started taking anti-TB drugs in late 2020 were excluded from further analysis. Among the 272 patients, 91 (33.5%) completed treatment, 1 (0.4%) was LTFU, and 62 (22.8%) died, including 18 (6.6%) who died after discharge; 118 (43.4%) had unknown outcomes (Table 3). 

We performed univariate (Appendix Table 5) and multivariate (Table 5) analyses of odds for postdischarge death. Multivariate analysis identified that patients with unknown BCG vaccination status (aOR 5.38 [95% CI 1.07–27.07]) and those with clinical findings during hospitalization such as hydrocephalus (aOR 18.97 [95% CI 2.68–134.38]) and tuberculoma (aOR 8.78 [95% CI 1.10–70.39]) had increased odds of postdischarge death. Among patients with hydrocephalus, the absence of neurosurgical intervention was associated with increased odds of postdischarge death (aOR 11.06 [95% CI 1.61–76.12]), adjusted for age, sex, and TBM staging.

**Table 5:** Multivariate logistic regression model for predictors of postdischarge death, tracked until the end of tuberculous meningitis treatment in children treated for TBM at Hasan Sadikin Hospital, Bandung, Indonesia, 2011–2020*†

Variable	Died‡§	Alive‡	Crude OR (95% CI)	p value	aOR (95% CI)	p value
No. cases	18	91				
Age group, y						
<2	3 (16.7)	26 (28.6)	0.65 (0.15–2.86)	0.573	0.13 (0.01–1.12)	0.064
2–4	6 (33.3)	9 (9.9)	3.78 (0.98–14.56)	0.054	1.60 (0.26–9.86)	0.610
5–9	3 (16.7)	22 (24.2)	0.77 (0.17–3.41)	0.734	0.23 (0.03–1.75)	0.156
10–14	6 (33.3)	34 (37.4)	Referent		Referent	
Sex						
M	10 (55.6)	39 (42.9)	1.67 (0.60–4.61)	0.325	3.43 (0.76–15.45)	0.109
F	8 (44.4)	52 (57.1)	Referent		Referent	
TBM stage¶						
Stage I or II	7 (38.9)	67 (73.6)	Referent		Referent	
Stage III	11 (61.1)	24 (26.4)	4.39 (1.53–12.6)	0.006	2.31 (0.56–9.54)	0.247
Known BCG vaccination						
No	7 (38.9)	15 (16.5)	3.22 (1.08–9.66)	0.037	5.38 (1.07–27.07)	0.041
Yes	11 (61.1)	76 (83.5)	Referent			
Hydrocephalus on CT						
No	3 (16.7)	66 (72.5)	Referent		Referent	
Yes	13 (72.2)	23 (25.3)	12.43 (3.25–47.59)	<0.001	18.97 (2.68–134.38)	0.003
Unknown	2 (11.1)	2 (2.2)	22.00 (2.26–214.23)	0.008	17.85 (1.30–245.49)	0.031
Tuberculoma on CT#						
No	12 (66.7)	85 (93.4)	Referent		Referent	
Yes	4 (22.2)	4 (4.4)	7.08 (1.56–32.13)	0.011	8.78 (1.10–70.39)	0.041
Positive TST						
No	10 (55.6)	76 (83.5)	Referent		Referent	
Yes	8 (44.4)	15 (16.5)	4.05 (1.37–11.96)	0.011	4.79 (0.96–24.05)	0.057

*Data are no. (%) except as indicated. aOR, adjusted odds ratio; BCG, bacillus Calmette-Guérin; CT, computed tomography; GCS, Glasgow Coma Scale; TBM, tuberculous meningitis; TST, tuberculin skin test. †The goodness-of-fit of the model using Hosmer-Lemeshow test was p = 0.877. The performance of the model using the area under the receiver operating characteristic curve was 0.91 (95% CI 0.85–0.97). ‡Including patients who were tracked until death or treatment completion, and excluding patients who were lost to follow-up and with unknown treatment outcomes. §Positive TST and motor disorders were associated with higher odds of postdischarge death in univariate analysis but did not remain significant in multivariate analysis. Signs of raised intracranial pressure with hydrocephalus as well as GCS score with TBM staging had the likelihood of collinearity; therefore, only hydrocephalus and TBM staging were included in the final multivariate model. In our subgroup analysis among children aged <5 y, no additional independent predictors of postdischarge death were observed. ¶Stage I TBM was defined as GCS of 15 with no focal neurologic signs, stage II TBM as GCS of 11–14 or 15 with focal neurologic signs, and stage III TBM as GCS ≤10 (*20*). Patients with stages I and II TBM were combined in the analysis because there were no patients with TBM stage I died after hospital discharge. #Because of the redundancy with the variable “unknown status of hydrocephalus,” the degree of freedom for the variable “unknown status of tuberculoma” was reduced.

### Neurologic Sequelae

Among 91 survivors who completed treatment, 58 (63.7%) had good recovery without neurologic sequelae and 33 (36.3%) had severe neurologic sequelae (Table 3). Of patients with severe neurologic sequelae, 22 (66.7%) had motor disorders, 9 (27.3%) had epileptic seizures, 7 (21.2%) had neurodevelopmental delay, 3 (9.1%) had visual impairment, and 3 (9.1%) had hearing impairment. Neurologic sequelae were observed in 23% of patients diagnosed with TBM at stage I, 31% at stage II, and 58% at stage III. 

We performed univariate (Appendix Table 6) and multivariate (Table 6) analyses of odds for neurologic sequelae. In multivariate analysis, factors associated with higher odds of severe neurologic sequelae were baseline temperature >38°C (aOR 6.68 [95% CI 1.55–28.85]), stage III TBM (aOR 5.65 [95% CI 1.21–26.43]), and motor deficits at baseline (aOR 3.64 [95% CI 1.19–11.16]). 

**Table 6:** Multivariate logistic regression model for predictors of severe neurologic sequelae at treatment completion in children treated for TBM at Hasan Sadikin Hospital, Bandung, Indonesia, 2011–2020*†

Variable	Neurologic sequelae		Crude OR (95% CI)	p value	aOR (95% CI)	p value
	Yes‡§	No‡				
Cases, no.	33	58				
Age group, y						
<2	13 (39.4)	13 (22.4)	2.78 (0.94–8.20)	0.064	2.59 (0.67–10.00)	0.166
2–4	2 (6.1)	7 (12.1)	0.79 (0.14–4.55)	0.795	0.97 (0.13–7.28)	0.974
5–9	9 (27.3)	13 (22.4)	1.92 (0.61–6.02)	0.261	1.32 (0.34–5.07)	0.684
10–14	9 (27.3)	25 (43.1)	Referent		Referent	
Sex						
M	12 (36.4)	27 (46.6)	0.66 (0.27–1.58)	0.346	0.48 (0.16–1.45)	0.191
F	21 (63.6)	31 (53.4)	Referent		Referent	
TBM stage¶						
Stage I	5 (15.2)	17 (29.3)	Referent		Referent	
Stage II	14 (42.4)	31 (53.4)	1.53 (0.47–5.00)	0.476	1.83 (0.43–7.75)	0.410
Stage III	14 (42.4)	10 (17.2)	4.76 (1.32–17.22)	0.017	5.65 (1.21–26.43)	0.028
Baseline temperature >38°C						
No	23 (69.7)	53 (91.4)	Referent		Referent	
Yes	10 (30.3)	5 (8.6)	4.61 (1.42–14.99)	0.011	6.68 (1.55–28.85)	0.011
Motor deficit at baseline						
No	8 (24.2)	27 (46.6)	Referent		Referent	
Yes	24 (72.7)	23 (39.7)	3.52 (1.33–9.33)	0.011	3.64 (1.19–11.16)	0.024
Unknown	1 (3.0)	8 (13.8)	0.42 (0.05–3.90)	0.447	0.39 (0.03–4.58)	0.452

*Values are no. (%) except as indicated. aOR, adjusted odds ratio; TB, tuberculosis; TBM, tuberculous meningitis. †The goodness-of-fit of the model using Hosmer-Lemeshow test was p = 0.473. The performance of the model using the area under the receiver operating characteristic curve was 0.80 (95% CI 0.70–0.90). ‡Including patients who were tracked until treatment completion, and excluding those who died, who were lost to follow-up and with unknown treatment outcomes (which represents a large percentage of the cohort (n = 118, 43.3%). Neurologic sequelae were defined as any motor, hearing, visual or neurodevelopmental impairment that appeared during the illness and persisted through treatment completion. §Suggestive TB through chest radiography was associated with an increased odd of neurologic sequelae in univariate analysis but did not remain significant in multivariate analysis. In our subgroup analysis among children aged <5 y, no additional independent predictors for neurologic sequelae were found. ¶Stage I TBM was defined as Glasgow Coma Scale (GCS) of 15 with no focal neurologic signs, stage II TBM as GCS of 11–14 or 15 with focal neurologic signs, and stage III TBM as GCS ≤10 (*20*).

## Discussion

We present important information from Indonesia about the high rates of neurologic sequelae and death in children with TBM, even when standard therapy has been provided. In TBM, treatment response is often judged by early morbidity, mortality, and relapse rates (*21*). Our overall case-fatality rate for childhood TBM (22.8%) is within the global estimates reported in a recent meta-analysis (19.3% [95% CI 14.0%–26.1%]) (*2*) but is lower than that reported in the same setting during 2007–2010 (34.4%) (*14*). The high proportion of unknown treatment outcomes in this study (43%) is unfortunate but comparable to a previous report in our hospital during 2007–2010 (45%), even after phone calls and home visits had been made (*14*). Considering the increased likelihood of death in patients with unknown outcomes after hospital discharge, the case-fatality rate recorded is probably an underestimate.

A diagnosis of TBM alone has been associated with an increased risk for childhood death compared with other types of TB (*22*), and this risk may be exacerbated by specific risk factors identified in this study. TBM diagnosis in stage II or III, hydrocephalus, and seizures are not surprising risk factors for death because they reflect more advanced disease. Neurosurgical complications (e.g., shunt blockage or infections) may have contributed to poor outcomes, but we believe the effect was minimal because the postdischarge death rate was significantly reduced with neurosurgery. The association of tuberculoma on baseline CT with postdischarge death might be related to a paradoxical worsening of tuberculomas during treatment (*23*). For male sex and low-income parents, their associations with in-hospital death are unclear but could be related to biologic factors (particularly for sex differences) or largely attributed to socioeconomic and cultural determinants (*24*).

This study confirms that TBM mainly affects young children (*8*), illustrated by 54% of our patients being <5 years of age. The high proportions of altered consciousness and seizures at admission suggest that these symptoms are the main reasons for clinicians to suspect childhood TBM. This finding raises important issues about training of healthcare providers to improve their ability to recognize and diagnose the disease (*25*). In addition, increasing community awareness of the signs and symptoms of TBM by including enhanced messaging in existing TB advocacy materials has the potential to improve early recognition of childhood TBM (*25*).

The difficulty of early diagnosis is confirmed by the fact that nearly 80% of our patients had stage II or III TBM at admission. This high proportion of patients with advanced disease at admission is supported by various studies from high TB incidence countries in Asia and Africa (*11*,*14*,*26*,*27*), and only slightly reduced in low TB incidence countries in Europe, where 66% of the patients have stage II or III TBM at admission (*28*). In many cases, nonspecific symptoms such as fever, headache, and vomiting are often wrongly interpreted, and other systemic symptoms such as cough, weight loss, and night sweats may be suggestive of TB but are also nonspecific (*18*).

The high risk for death in patients with unknown BCG vaccination status highlights the importance of better TB prevention. In young children, BCG vaccination has consistently shown protection against miliary TB and TBM (*29*–*31*) for >10 years after vaccination (*29*). The global shortage of BCG vaccine since 2013, particularly in countries where it was procured through UNICEF (*32*), has led to an alarming increase in the number of hospital admissions for childhood TBM (*33*). In Indonesia, where BCG vaccine is recommended at birth for all infants and annual coverage has been an estimated ≈90% since 2011 (*34*), this shortage has also been experienced, although the vaccine supply depends largely on domestic production by Biofarma, a state-owned vaccine manufacturer (*32*). In addition, among children with prolonged exposure to *M. tuberculosis*, protection with BCG vaccination alone is unlikely to be sufficient. Without early initiation of preventive therapy, the risk for TB disease development among exposed young children and infants is very high (*35*), but data on preventive treatment in our patients with known TB contact history were unavailable. Taken together, aside from improving BCG vaccination coverage, it is important to reduce TB transmission in children through contact investigation, coupled with preventive therapy among exposed children.

Neurologic sequelae occurred mostly in our patients who had stage III TBM at admission (58%), a higher rate than for those in stage I (23%) and II (31%). A meta-analysis in children with TBM confirms this upward trend with pooled estimates of 27% in stage I, 41% in stage II, and 70% in stage III (*2*). Recent studies also reported an increase in neurologic sequelae among children with stage II or III TBM (*36*,*37*). In children in South Africa with TBM, severe neurologic sequelae and death were significantly associated with cerebral infarctions (*11*); we did not find this association in our study. A high proportion of patients had hemiparesis or quadriparesis at admission in this study (55%), comparable to that reported in South Africa (62.1%) (*11*), but few patients had cerebral infarcts on brain CT (10%). This finding is difficult to explain but is likely attributable to the low sensitivity of early infarct detection with noncontrast CT as commonly used in the study.

Given the substantial levels of neurologic sequelae and death associated with childhood TBM, the current standard care for childhood TBM clearly remains suboptimal. New diagnostic strategies should be tested in future clinical trials because of the poor sensitivity, specificity, or both of available laboratory and clinical diagnostic tools (*38*). For TBM treatment, future research should explore the use of intensified antimicrobial therapy that contains high-dose rifampin and other anti-TB drugs with better CSF penetration and bactericidal activity (*39*). On the basis of observational data among children in South Africa, a 6-month intensified TBM treatment regimen with isoniazid, rifampin, and ethionamide at 20 mg/kg/day and pyrazinamide at 40 mg/kg/day was reported to be safe and effective, with lower case-fatality rates ranging from 4%–14% (*11*,*12*,*40*). This short-course, high-dose therapy has recently been added by WHO as an alternative treatment option for childhood TBM (*41*). Suboptimal plasma and CSF concentrations with standard doses of oral rifampin at 10–20 mg/kg/day in children with TBM have also been reported in recent pharmacokinetic studies (*42**,**43*), advocating the use of higher rifampin doses with further efficacy and safety evaluations.

Minimizing damaging immunologic responses leading to neurologic complications by using antiinflammatory drugs such as aspirin, thalidomide, and specific tumor necrosis factor α antibodies (e.g., infliximab) also warrants further investigations (*10*,*44*–*46*), particularly for paradoxical TBM reactions and potentially also for TBM in general. There is no evidence that corticosteroids (the mainstay of host-directed therapy) reduce neurologic sequelae although they do improve the TBM survival rate (*47*). Therefore, optimization of anti-TB drug dosing and consideration of immunomodulatory therapy beyond corticosteroids are required to improve childhood TBM treatment outcomes (*9*,*46*). Moreover, understanding the disease pathogenesis pathways of childhood TBM, particularly in the cerebral inflammatory response, is likely to offer valuable insights into potential targets for new treatment interventions (*48*,*49*).

The main limitation of our study is that, although most of the essential information recommended for TBM research was available (*50*), the retrospective nature of the study did not provide us with complete records on all key variables, especially longer-term outcome. Our dataset did not contain information on the drug-susceptibility pattern of the source case and was unable to reliably distinguish a contact history with an infectious drug-susceptible or drug-resistant TB case. This limitation may have led to underdiagnosis of drug-resistant TB disease, resulting in inappropriate antimicrobial therapy that may have contributed to poor outcomes. However, drug-resistance rates are not known to be high in the study population, an estimated 2.4% of multidrug-resistant TB among new cases in Indonesia (*1*), limiting the likely effect of inappropriate treatment of drug-resistant disease. In addition, the frequency of total neurologic sequelae at treatment completion might be underestimated in this study, given that mild to moderate sequelae were not tested or recorded in the database. Despite its limitations, this study provides one of the largest child TBM cohorts ever described globally outside of South Africa (*11*), and includes a wide range of variables in the analysis.

In conclusion, childhood TBM in Indonesia causes substantial neurologic sequelae and death, despite standard treatment. Several predictors of in-hospital death, postdischarge death, and neurologic sequelae have been identified for further development of early and tailored interventions to optimize care in this population. This study emphasizes the importance of improved early diagnosis, better TB prevention beyond BCG vaccination, and optimizing TBM management strategies, including antimicrobial and supportive therapy.

Appendix Additional information about treatment outcomes of childhood tuberculous meningitis in a real-world retrospective cohort, Bandung, Indonesia.
