# Supplementary material for: Treatment Outcomes of Childhood Tuberculous Meningitis in a Real-World Retrospective Cohort, Bandung, Indonesia
Source: Emerg Infect Dis. 2022 Mar;28(3):660–71. doi: 10.3201/eid2803.212230 (PMC8888221; doi:10.3201/eid2803.212230)
Supplement: Appendix — Additional information about treatment outcomes of childhood tuberculous meningitis in a real-world retrospective cohort, Bandung, Indonesia. [file 21-2230-Techapp-s1.pdf]

Article DOI: https://doi.org/10.3201/eid2803.212230

Treatment Outcomes of Childhood Tuberculous Meningitis in a Real-World Retrospective Cohort, Bandung, Indonesia

Appendix

Assessments of neurologic sequelae in children with tuberculous meningitis at treatment completion

In this study, neurologic sequelae were defined as any motor, hearing, visual or neurodevelopmental impairment that appeared during the illness and persisted through treatment completion. Only severe neurologic sequelae were recorded in the database. Mild to moderate sequelae were not routinely tested or recorded, and complete assessments were only performed if indicated or requested by the attending physicians. Generally, hearing function was assessed using the Brainstem Auditory Evoked Response (BAER) method. Degree of hearing loss was determined based on the Pure Tone Average (PTA). Visual examinations consisted of a visual acuity test using Cardiff Acuity Cards or the Snellen Chart, an extraocular muscle examination test, the anterior segment of the eyeball examination test using a slit lamp or a loop/magnifier 3D and a penlight, and the posterior segment of the eyeball/fundus examination test using direct and indirect ophthalmoscope. Neurodevelopmental function in children aged ≤8 years was assessed using the Griffiths General Developmental Quotient. In children aged >8 years, neurodevelopmental function was assessed using the Wechsler Intelligence Scale for Children (WISC). Gross motor function was assessed using the Growth Motor Functional Measurement (GMFM). Detailed methods and classifications have been described elsewhere (*1*).

Case presentation of a TBM patient with SARS-CoV-2 coinfection

A 6-month-old boy (weight: 5.3 kg, height: 60 cm, head circumference: 46 cm) presented at Hasan Sadikin Hospital in April 2020, with a 10-day history of fever prior to admission. He also had seizures, quadriparesis and vomiting, but no other symptoms suggestive of TBM were

reported at presentation. Prior to admission, he had a diagnosis of congenital hydrocephalus and underwent a ventriculoperitoneal shunt in another hospital. He had an unknown history of recent close contact with a TB patient, had been vaccinated with BCG, was severely malnourished and had a GCS score of 15 with focal neurologic deficits. In cerebrospinal fluid (CSF) analysis, he had pleocytosis of 355 cells/µL, abnormal protein concentration of 848 mg/dL, lymphocytic predominance of 59.7%, CSF to blood glucose ratio of 30% and smear-negative for acid fast-bacilli (AFB). He had a negative result on tuberculin skin test and had bronchopneumonia dextra on chest radiography. Mycobacterial cultures from CSF and gastric lavage were negative, AFB smear microscopy was positive from gastric lavage, and *M. tuberculosis* sensitive to rifampicin was identified through GeneXpert MTB/RIF assay from gastric lavage. Brain computed tomography scan results showed communicating hydrocephalus, with negative signs for basal meningeal enhancement, infarct or tuberculoma. He was diagnosed with probable TBM at stage II, and was treated with daily oral isoniazid at 10 mg/kg, rifampicin at 15 mg/kg, pyrazinamide at 35 mg/kg and ethambutol at 20 mg/kg, for a 2-month intensive phase, and followed by a 10-month continuation therapy with isoniazid and rifampicin at the same doses. Adjunctive oral prednisone at 2 mg/kg was given for the first 4 weeks of treatment. During hospitalization, facility-based directly observed treatment was used by the treated physician or nurses to administer the drugs. He was discharged after 20 days of hospitalization, with existing complications of hearing impairment and motor disorders.

After discharge, TBM treatment with first-line anti-TB drugs was continued for up to 12 months, and he was followed up monthly at Hasan Sadikin Hospital. During the 8-month follow-up, he was tested positive for coronavirus SARS-CoV-2 infection by real-time reverse transcription-polymerase chain reaction swab test (RdRp- and E-genes), with no specific symptoms for COVID-19. After 1 day of hospitalization, he was discharged and advised to self-isolate for 3 weeks. No antiretroviral drugs were administered. After 3 weeks, he was confirmed negative from SARS-CoV-2. At treatment completion, TBM symptoms of fever and seizures were not present. Bodyweight, height and head circumference had increased to 9.8 kg, 81 cm and 52 cm, respectively. Neurologic sequelae of motor disorders persisted through treatment completion. He was considered a failure to thrive, with only being able to tilt his body to the right and unable to babble.

Appendix Table 1. Diagnostic certainty of tuberculous meningitis using uniform case definition criteria by Marais et al (*2*).

Characteristic	Score	Total patients (n=283)	Possible/ probable TBM (n=232)
Clinical criteria [maximum category score = 6]			
Symptoms duration of more than 5 days	4	234 (82.7)	191 (82.3)
Systemic symptoms suggestive of TB (one or more of the following: weight loss / poor weight gain, night sweats, or persistent cough for more than 2 weeks)	2	263 (92.9)	216 (93.1)
History of recent (within past year) close contact with an individual with pulmonary TB or a positive TST or IGRA (only in children under 10 years of age)	2	114 (40.3)	94 (40.5)
Focal neurologic deficit (excluding cranial nerve palsies)	1	222 (78.4)	179 (77.2)
Cranial nerve palsy	1	48 (17.0)	36 (15.5)
Altered consciousness	1	211 (74.6)	169 (72.8)
CSF criteria [maximum category score = 4)			
Clear appearance	1	276 (97.5)	225 (97.0)
Leucocyte cells: 10-500 per µL	1	212 (74.9)	172 (74.1)
Lymphocytic predominance of >50%	1	225 (79.5)	182 (78.4)
Protein concentration >100 mg/dL	1	143 (50.5)	115 (49.6)
CSF / plasma glucose ratio of <50% or an absolute CSF glucose concentration <40 mg/dL	1	142 (50.0)	115 (49.6)
Cerebral imaging criteria [maximum category score = 6]			
Hydrocephalus	1	103 (41.2)	84 (36.2)
Basal meningeal enhancement	2	131 (46.3)	104 (44.8)
Tuberculoma	2	31 (11.0)	22 (9.5)
Infarct	1	25 (8.8)	21 (9.1)
Pre-contrast basal hyperdensity	2	-	-
Evidence of TB elsewhere [maximum category score = 4]			
Chest radiography suggestive of active TB			
Miliary TB	4	19 (6.7)	16 (6.9)
Other signs of TB	2	128 (45.2)	103 (44.4)
CT / MRI / USG evidence of TB outside the CNS	2	-	-
AFB identified or *M. tuberculosis* cultured from another source (sputum, lymph node, gastric aspirates, urine or blood culture)	4	65 (23.0)	37 (15.9)
Positive commercial *M. tuberculosis* nucleic acid amplification test (NAAT) from non-CSF specimen	4	76 (26.8)	38 (16.4)
Definite TBM (AFB seen on CSF microscopy, *M. tb* cultured from CSF, or *M. tb* detected through GeneXpert test)		51 (18.0)	
Probable TBM (total score of ≥12 when neuroimaging available or total score of ≥10 when neuroimaging was unavailable)		178 (62.9)	
Possible TBM (total score of 6-11 when neuroimaging available, or total score of 6-9 when neuroimaging was unavailable)		54 (19.1)	

Appendix Table 2. Operational definition for variables used in this study

Variable	Definition
Children	Individuals aged <15 years at diagnosis were defined as children, and were generally categorized by three age bands (0-4 years, 5-9 years and 10-14 years) as recommended by the WHO (*3*). An age group of less than 2 years was added given the high risk of severe progression to miliary and meningitis TB following infection with *M. tuberculosis* (*4*).
Malnourished	Children aged <5 years with weight-for-age or height-for-age Z-scores <-2 standard deviations, or children aged ≥5 years with height-for-age or BMI-for-age Z-scores <-2 standard deviation (*5*,*6*).
Known TB contact history	A patient who had close contact history with an infectious TB patient within the past year before hospital admission.
Known BCG vaccination	A documented BCG vaccination history in the immunization records book (*Buku Kesehatan Ibu dan Anak)* at the time of hospital admission, and/or the presence of a BCG scar in the deltoid part of the upper arm.
Definite TBM	Microbiological confirmation from CSF examination, including AFB smear microscopy, mycobacterial culture or GeneXpert MTB/RIF testing (*2*).
Probable TBM	A total diagnostic score of ≥12 when neuroimaging was available, or ≥10 when neuroimaging was unavailable (*2*).
Possible TBM	A total diagnostic score of 6-11 when neuroimaging was available, or 6-9 when neuroimaging was unavailable (*2*).
TBM stage I	Glasgow Coma Scale (GCS) scores of 15 without focal neurologic signs (*7*).
TBM stage II	GCS scores of 11-14 or 15 with focal neurologic signs (*7*).
TBM stage III	GCS scores of ≤10 (*7*).

Variable	Definition
Cranial nerve palsy	Cranial nerve palsy was characterized by a decreased or complete loss function of one or more cranial nerves.
Motor deficits	Motor deficits included hemiparesis, quadriparesis and diplegia.
Signs of upper neuron lesion	Signs of upper motor neuron lesion included muscle weakness, hypertonus, clonus, hyperreflexia and the presence of pathological reflex.
Signs raised intracranial pressure	In physical examination, signs of raised intracranial pressure could include cranial nerve IV palsy, excessive headache/vomiting, papilledema, bulging fontanelle, sunsetting sign, etc. The diagnosis could also be made through cerebral imaging.
Hydrocephalus on neuroimaging	Enlargement of the ventricles with the compression of sulci and gyri and enlargement of temporal horn >2 mm or frontal horn-to-internal diameter ratio >0.5, supported by ballooning of the frontal horns, transependymal edema, Evans ratio of >0.3, and sagittal bowing of corpus callosum (*8*).
Suggestive of TB from chest radiography	Chest radiographic findings suggestive of TB included mediastinal/hilar lymphadenopathy, segmental infiltration and/or collapse, pleural effusion, cavitation, and signs of miliary TB.
Positive tuberculin skin test	Induration of ≥10 mm in Mantoux skin test, or ≥5 mm in patients with severe malnutrition or HIV-infected children.
AFB smear positive	The specimen from cerebrospinal fluid, sputum, gastric lavage or other body materials noted as at least +1 for acid-fast bacilli (AFB) on microscopy using Ziehl-Neelsen stain.
Anti-TB drug-induced hepatotoxicity	Anti-TB drug-induced hepatotoxicity was defined according to the modified American Thoracic Society guidelines, developed internally by the Department of Child Health, Universitas Padjadjaran, as follows: an elevation of alanine aminotransferase (ALT) >3× the ULN with symptoms of hepatotoxicity, an elevation of ALT >5× the ULN without symptoms, normal baseline ALT with the presence of jaundice, anorexia, nausea and vomiting during treatment, or an increased in total bilirubin level >1.5 mg/dL (*9*,*10*).

Appendix Table 3. Symptoms of tuberculous meningitis at presentation stratified by disease staging, in children with tuberculous meningitis treated at Hasan Sadikin Hospital, Bandung, Indonesia, 2011–2020

	Stage I		Stage II		Stage III	
Symptom	N*	Value	N*	Value	N*	Value
Fever	57	46 (80.7)	131	122 (93.1)	95	82 (86.3)
Severe headache	56	15 (26.8)	131	31 (23.7)	91	15 (15.8)
Muscle weakness	57	12 (21.1)	129	36 (27.5)	92	25 (26.3)
Altered consciousness	57	18 (31.6)	131	110 (84.0)	95	83 (87.4)
Seizures	57	28 (49.1)	131	63 (48.1)	95	64 (67.4)
Shortness of breath	57	13 (22.8)	130	14 (10.7)	93	17 (17.9)
Persistent cough	57	21 (36.8)	131	41 (31.3)	94	33 (35.1)
Poor weight gain / weight loss	57	16 (28.1)	130	44 (33.6)	92	45 (47.4)

Data are presented as number (n) with percentages (%). *: Number of total patients for which data were available. Stage I TBM was defined as Glasgow Coma Scale (GCS) of 15 with no focal neurologic signs, stage II TBM as GCS of 11-14 or 15 with focal neurologic signs, and stage III TBM as GCS ≤10 (7).

Appendix Table 4. Univariate Cox proportional-hazards regression model for factors associated with in-hospital death in children treated for tuberculous meningitis at Hasan Sadikin Hospital, Bandung, Indonesia, 2011–2020

Patient characteristics	Dead (n=44)	Alive (n=231)	cHR (95% CI)	p-value
Year of diagnosis (median (IQR))[a,b]	2018 (2015-2019)	2016 (2014-2019)	1.11 (0.98-1.25)	0.087
Year of diagnosis[a,b]				
2011-2015	11 (25.0)	91 (39.4)	0.55 (0.28-1.08)	0.083
2016-2020	33 (75.0)	140 (60.6)	1.00	
Age, years (median (IQR))	4.0 (1.5-10.7)	1.0 (4.0-10.0)	1.00 (0.94-1.07)	0.876
Age[a]				
<2 years	13 (29.5)	78 (33.8)	0.78 (0.37-1.67)	0.527
2-4 years	11 (25.0)	47 (20.3)	1.04 (0.47-2.29)	0.992
5-9 years	6 (13.6)	43 (18.6)	0.65 (0.25-1.70)	0.384
10-14 years	14 (31.8)	63 (27.3)	1.00	
Sex[a]				
Male	29 (65.9)	118 (51.1)	1.72 (0.92-3.20)	0.089
Female	15 (34.1)	113 (48.9)	1.00	
Parent’s last education				
Elementary	4 (9.1)	38 (16.5)	0.83 (0.15-4.53)	0.829
Junior high school	16 (36.4)	64 (27.7)	1.88 (0.43-8.16)	0.401
Senior high school	21 (47.7)	110 (47.6)	1.47 (0.34-6.25)	0.605
University	2 (4.5)	15 (6.5)	1.00	
Parent’s monthly income[a]				
USD ≤140,00	33 (75.0)	136 (58.9)	2.79 (1.17-6.67)	0.021
USD >140,00	6 (13.6)	74 (32.0)	1.00	
Area of living				
Urban	17 (38.6)	98 (42.4)	1.00	

Patient characteristics	Dead (n=44)	Alive (n=231)	cHR (95% CI)	p-value
Rural	25 (56.8)	126 (54.5)	1.13 (0.61-2.10)	0.692
Weight-for-age Z-score				
≥-2 (normal)	19 (43.2)	82 (35.5)	1.00	
<-2 (underweight)	17 (38.6)	103 (44.6)	0.72 (0.38-1.39)	0.336
Height-for-age Z-score				
≥-2 (normal)	24 (54.5)	141 (61.0)	1.00	
<-2 (stunted)	20 (45.5)	90 (39.0)	1.25 (0.69-2.26)	0.460
Weight-for-height Z-score				
≥-2 (normal)	21 (47.7)	115 (49.8)	1.00	
<-2 (wasted)	23 (52.3)	116 (50.2)	1.06 (0.59-1.92)	0.837
BMI-for-age Z-score				
≥-2 (normal)	22 (50.0)	113 (48.9)	1.00	
<-2 (low BMI)	22 (50.0)	118 (51.1)	0.96 (0.53-1.72)	0.881
Nutritional status[§]				
Normal	16 (36.4)	81 (35.1)	1.00	
Moderate malnutrition	10 (22.7)	60 (26.0)	0.82 (0.37-1.82)	0.634
Severe malnutrition	18 (40.9)	90 (39.0)	1.00 (0.51-1.95)	0.991
Known BCG vaccination[a]				
No	15 (34.1)	44 (19.0)	2.01 (1.08-3.76)	0.028
Yes	29 (65.9)	187 (81.0)	1.00	
Known TB contact history[a]				
No	27 (61.4)	176 (76.2)	1.00	
Yes	17 (38.6)	55 (23.8)	1.83 (1.00-3.35)	0.051
Known HIV status[a,e]				
No/unknown	41 (93.2)	230 (99.6)	1.00	
Yes	3 (6.8)	1 (0.4)	6.46 (1.99-20.92)	0.002
TBM category and stage				
TBM category[¥]				
Definite TBM	9 (20.5)	39 (16.9)	1.00	
Probable TBM	25 (56.8)	147 (63.6)	0.72 (0.34-1.55)	0.406
Possible TBM	10 (22.7)	45 (19.5)	0.93 (0.38-2.29)	0.857
TBM stage[¶,a,c]				
Stage I	2 (4.5)	54 (23.4)	1.00	
Stage II	15 (34.1)	111 (48.1)	3.53 (0.81-15.44)	0.094
Stage III	27 (61.4)	66 (28.6)	9.16 (2.18-38.51)	0.003
GCS (median (IQR))[a,c]	10 (9-12)	12 (11-15)	0.80 (0.72-0.88)	<0.001
Presenting symptoms				
Fever				
No	6 (13.6)	27 (11.7)	1.00	
Yes	38 (86.4)	204 (88.3)	0.87 (0.37-2.07)	0.759
Severe headache				
No	31 (70.5)	179 (77.5)	1.00	
Yes	11 (25.0)	49 (21.2)	1.30 (0.66-2.60)	0.448
Muscle weakness				
No	33 (75.0)	169 (73.2)	1.00	
Yes	10 (22.7)	58 (25.1)	0.87 (0.43-1.76)	0.700
Altered consciousness				
No	7 (15.9)	64 (27.7)	1.00	
Yes	37 (84.1)	167 (72.3)	1.91 (0.85-4.30)	0.115
Seizures[a]				
No	13 (29.5)	112 (49.5)	1.00	
Yes	31 (70.5)	119 (51.5)	2.09 (1.09-3.99)	0.026
Shortness of breath[a]				
No	32 (72.7)	196 (84.8)	1.00	
Yes	11 (25.0)	33 (14.3)	1.81 (0.91-3.59)	0.090
Persistent cough				
No	27 (61.4)	155 (67.1)	1.00	
Yes	16 (36.4)	76 (32.9)	1.20 (0.64-2.22)	0.567
Poor weight gain / weight loss				
No	26 (59.1)	142 (61.5)	1.00	
Yes	17 (38.6)	86 (37.2)	1.06 (0.57-1.95)	0.861
Duration of symptoms				
0-7 days	15 (34.1)	84 (36.4)	1.00	
8-14 days	25 (56.8)	119 (51.5)	1.16 (0.61-2.19)	0.658
>14 days	1 (2.3)	17 (7.4)	0.34 (0.04-2.58)	0.298
Examination findings at baseline				
Body temperature				
<38 °C	33 (75.0)	181 (78.4)	1.00	
≥38 °C	10 (22.7)	50 (21.6)	1.06 (0.52-2.16)	0.865

Patient characteristics	Dead (n=44)	Alive (n=231)	cHR (95% CI)	p-value
Respiration rate				
<25/min	11 (25.0)	78 (33.8)	1.00	
≥25/min	32 (72.7)	151 (65.4)	1.43 (0.72-2.84)	0.304
Involuntary movement				
No	36 (81.8)	203 (87.9)	1.00	
Yes	6 (13.6)	22 (9.5)	1.54 (0.65-3.65)	0.329
Cranial nerve palsies				
No	34 (77.3)	187 (81.0)	1.00	
Yes	8 (18.2)	40 (17.3)	1.14 (0.53-2.47)	0.736
Any type of motor deficit				
No	20 (45.5)	90 (39.0)	1.00	
Yes	21 (47.7)	124 (53.7)	0.82 (0.44-1.50)	0.515
Unequal pupils				
No	39 (88.6)	221 (95.7)	1.00	
Yes	3 (6.8)	6 (2.6)	2.49 (0.77-8.07)	0.127
Signs of upper motor neuron lesions[a]				
No	6 (13.6)	64 (27.7)	1.00	
Yes	36 (81.8)	150 (64.9)	2.46 (1.03-5.83)	0.042
Signs of raised intracranial pressure[a,d]				
No	25 (56.8)	203 (87.9)	1.00	
Yes	19 (43.2)	28 (12.1)	4.39 (2.41-7.97)	<0.001
CSF findings				
Leucocyte ≥10 cells/μL[a]				
No	14 (31.8)	48 (20.8)	1.00	
Yes	27 (61.4)	179 (77.5)	0.56 (0.29-1.06)	0.076
Leucocyte ≥100 cells/μL				
No	32 (72.7)	160 (69.3)	1.00	
Yes	9 (20.5)	67 (29.0)	0.71 (0.34-1.49)	0.370
Lymphocytic predominance >50%				
No	8 (18.2)	40 (17.3)	1.00	
Yes	34 (77.3)	185 (80.1)	0.93 (0.43-2.00)	0.846
Protein >100 mg/dL				
No	22 (50.0)	107 (46.3)	1.00	
Yes	20 (45.5)	119 (51.5)	0.82 (0.45-1.51)	0.532
Glucose <40 mg/dL				
No	22 (50.0)	133 (57.6)	1.00	
Yes	20 (45.5)	86 (37.2)	1.36 (0.74-2.49)	0.322
CSF/blood glucose ratio <50%				
No	15 (34.1)	93 (40.3)	1.00	
Yes	22 (50.0)	103 (44.6)	1.31 (0.68-2.53)	0.417
Radiological findings				
Chest radiography				
Normal	23 (52.3)	108 (46.8)	1.00	
Miliary TB	2 (4.5)	17 (7.4)	0.57 (0.13-2.42)	0.448
Other signs of TB	19 (43.2)	104 (45.0)	0.88 (0.48-1.62)	0.682
Hydrocephalus[a,d]				
No	12 (27.3)	133 (57.6)	1.00	
Yes	22 (50.0)	76 (32.9)	3.00 (1.48-6.05)	0.002
Neurosurgery in hydrocephalus patients[§§]				
No	13 (59.1)	40 (52.6)	1.25 (0.53-2.93)	0.604
Yes	9 (40.9)	36 (47.4)	1.00	
Basal meningeal enhancement				
No	14 (31.8)	101 (43.7)	1.00	
Yes	20 (45.5)	108 (46.8)	1.30 (0.66-2.58)	0.445
Cerebral infarct				
No	30 (68.2)	189 (81.8)	1.00	
Yes	4 (9.1)	20 (8.7)	1.23 (0.43-3.51)	0.692
Tuberculoma				
No	28 (63.6)	185 (80.1)	1.00	
Yes	6 (13.6)	24 (10.4)	1.59 (0.66-3.84)	0.302
At least 1 sign found on CT scan				
No	7 (15.9)	67 (29.0)	1.00	
Yes	27 (61.4)	142 (61.5)	1.76 (0.77-4.04)	0.183
Bacteriological findings				
TST positive				
No	38 (86.4)	176 (76.2)	1.00	
Yes	6 (13.6)	55 (23.8)	0.54 (0.23-1.28)	0.164
GeneXpert MTB/RIF testing				
Negative	25 (56.8)	135 (58.4)	1.00	

Patient characteristics	Dead (n=44)	Alive (n=231)	cHR (95% CI)	p-value
M.tb identified from CSF	8 (18.2)	37 (16.0)	1.21 (0.55-2.69)	0.632
M.tb identified from non-CSF	5 (11.4)	34 (14.7)	0.81 (0.31-2.13)	0.675
AFB smear microscopy				
Negative	33 (75.0)	184 (79.7)	1.00	
Positive from CSF	2 (4.5)	4 (1.7)	2.44 (0.59-10.19)	0.220
Positive from non-CSF	7 (15.9)	34 (14.7)	1.15 (0.51-2.60)	0.737
M. tb cultured from any source				
No	36 (81.8)	200 (86.6)	1.00	
Yes	2 (4.5)	21 (9.1)	0.56 (0.13-2.34)	0.429
In-hospital complications				
Motor disorders				
No	31 (70.5)	142 (61.5)	1.00	
Yes	12 (27.3)	89 (38.5)	0.66 (0.34-1.28)	0.216
Visual impairment				
No	42 (95.5)	215 (93.1)	1.00	
Yes	1 (2.3)	16 (6.9)	0.33 (0.05-2.40)	0.275
Hearing impairment				
No	41 (93.2)	221 (95.7)	1.00	
Yes	2 (4.5)	10 (4.3)	1.13 (0.27-4.69)	0.862
Neurodevelopmental delay				
No	37 (84.1)	200 (86.6)	1.00	
Yes	6 (13.6)	31 (13.4)	1.05 (0.44-2.49)	0.913
Epileptic seizures				
No	39 (88.6)	217 (93.9)	1.00	
Yes	5 (11.4)	14 (6.1)	1.81 (0.71-4.59)	0.212
Anti-TB drug-induced hepatotoxicity				
No	39 (88.6)	209 (90.5)	1.00	
Yes	5 (11.4)	22 (9.5)	1.21 (0.47-3.06)	0.693
Others				
Oral corticosteroid				
No	2 (4.5)	10 (4.3)	1.00	
Yes	40 (90.9)	214 (92.6)	0.94 (0.23-3.88)	0.931

cHR: crude hazard ratio, AFB: acid-fast bacilli, BCG: Bacillus Calmette-Guerin, CSF: cerebrospinal fluid, CI: confidence interval, GCS: Glasgow Coma Scale, HIV: human immunodeficiency virus, IQR: interquartile rage, TB: tuberculosis, TBM: tuberculous meningitis, TST: tuberculin skin test.

[§]In children aged <5 years, moderate malnutrition was defined as weight-for-age or height-for-age Z-scores ≥-3 and <-2 standard deviations (SD), and severe malnutrition as weight-for-age or height-for-age Z-scores <-3 SD. In children aged 5-14 years, moderate malnutrition was defined as height-for-age or BMI-for-age Z-scores ≥-3 and <-2 SD, and severe malnutrition as height-for-age or BMI-for-age Z-scores <-3 SD (*5*).

[¥]Diagnostic score was assessed using a uniform case definition criteria for TBM, and was categorized as definite TBM (microbiologically proven from CSF examination), probable TBM (diagnostic score of ≥10 points when cerebral imaging is not available or ≥12 points when cerebral imaging is available), and possible TBM (diagnostic score of 6-9 points when cerebral imaging is not available or 6-11 points when cerebral imaging is available) (*2*).

[¶]Severity of TBM was classified according to the modified British Medical Research Council grading system as stage I (GCS of 15 with no focal neurologic signs), stage II (GCS of 11-14 or 15 with focal neurologic signs), or stage III (GCS ≤10) (7).

[§§]Analysis was only performed in patients with hydrocephalus

[a]Variables eligible for inclusion in multivariate analysis.

[b,c,d]Due to the likelihood of collinearity (e.g. TBM stage *vs.* GCS score and hydrocephalus *vs.* signs of raised intracranial pressure), only one of each of these variables was included during the development of the final multivariate model.

[e]Even though HIV coinfection was found to be significantly associated with in-hospital death in univariate analysis, we did not include this variable in multivariate analysis due to the selective HIV testing and a very low number of patients with HIV positive (n=4), which might limit the statistical power of the analysis.

Appendix Table 5. Univariate logistic regression model for predictors of post-discharge death, tracked until the end of tuberculous meningitis treatment in children treated for tuberculous meningitis at Hasan Sadikin Hospital, Bandung, Indonesia, 2011–2020

Patient characteristics	Dead (n=18)	Alive (n=91)	cOR (95% CI)	p-value
Year of diagnosis (median (IQR))	2017 (2015-2018)	2018 (2016-2019)	0.96 (0.76-1.20)	0.703
Year of diagnosis				
2011-2015	4 (22.2)	14 (15.4)	1.57 (0.45-5.48)	0.478
2016-2020	14 (77.8)	77 (84.6)	1.00	
Age, years (median (IQR))	4.0 (2.0-12.2)	7.0 (1.2-11.0)	0.98 (0.89-1.09)	0.752
Age[a]				
<2 years	3 (16.7)	26 (28.6)	0.65 (0.15-2.86)	0.573
2-4 years	6 (33.3)	9 (9.9)	3.78 (0.98-14.56)	0.054
5-9 years	3 (16.7)	22 (24.2)	0.77 (0.17-3.41)	0.734
10-14 years	6 (33.3)	34 (37.4)	1.00	
Sex[a]				
Male	10 (55.6)	39 (42.9)	1.67 (0.60-4.61)	0.325
Female	8 (44.4)	52 (57.1)	1.00	

Patient characteristics	Dead (n=18)	Alive (n=91)	cOR (95% CI)	p-value
Parent's last education				
Junior high school or lower	9 (50.0)	40 (44.0)	1.43 (0.51-4.05)	0.496
Senior high school or higher	8 (44.4)	51 (56.0)	1.00	
Parent's monthly income				
USD ≤140,00	12 (66.7)	52 (57.1)	1.96 (0.58-6.59)	0.276
USD >140,00	4 (22.2)	34 (37.4)	1.00	
Area of living				
Urban	10 (55.6)	40 (44.0)	1.00	
Rural	6 (33.3)	49 (53.8)	0.49 (0.16-1.46)	0.201
Weight-for-age Z-score				
≥-2 (normal)	6 (33.3)	34 (37.4)	1.00	
<-2 (underweight)	6 (33.3)	37 (40.7)	0.92 (0.27-3.12)	0.892
Height-for-age Z-score				
≥-2 (normal)	11 (61.1)	53 (58.2)	1.00	
<-2 (stunted)	7 (38.9)	38 (41.8)	0.89 (0.31-2.50)	0.821
Weight-for-height Z-score				
≥-2 (normal)	7 (38.9)	47 (51.6)	1.00	
<-2 (wasted)	11 (61.1)	44 (48.4)	1.67 (0.60-4.72)	0.326
BMI-for-age Z-score				
≥-2 (normal)	7 (38.9)	47 (51.6)	1.00	
<-2 (low BMI)	11 (61.1)	44 (48.4)	1.67 (0.60-4.72)	0.326
Nutritional status[§]				
Normal	6 (33.3)	30 (33.0)	1.00	
Moderate malnutrition	7 (38.9)	29 (31.9)	1.21 (0.36-4.02)	0.760
Severe malnutrition	5 (27.8)	32 (35.2)	0.78 (0.22-2.83)	0.707
Known BCG vaccination[a]				
No	7 (38.9)	15 (16.5)	3.22 (1.08-9.66)	0.037
Yes	11 (61.1)	76 (83.5)	1.00	
Known TB contact history				
No	13 (72.2)	68 (74.7)	1.00	
Yes	5 (27.8)	23 (25.3)	1.14 (0.37-3.54)	0.824
TBM category and stage				
TBM category[¥]				
Definite TBM	5 (27.8)	19 (20.9)	1.00	
Probable TBM	11 (61.1)	54 (59.3)	0.77 (0.24-2.52)	0.670
Possible TBM	2 (11.1)	18 (19.8)	0.42 (0.07-2.46)	0.338
TBM stage[¶,a,b]				
Stage I and II	7 (38.9)	67 (73.6)	1.00	
Stage III	11 (61.1)	24 (26.4)	4.39 (1.53-12.6)	0.006
GCS (median (IQR)) [a,b]	11 (10-12)	12 (11-14)	0.78 (0.64-0.96)	0.017
Presenting symptoms				
Fever				
No	2 (11.1)	7 (7.7)	1.00	
Yes	16 (88.9)	84 (92.3)	0.67 (0.13-3.51)	0.632
Severe headache				
No	13 (72.2)	59 (64.8)	1.00	
Yes	4 (22.2)	30 (33.0)	0.60 (0.18-2.02)	0.413
Muscle weakness				
No	11 (61.1)	65 (71.4)	1.00	
Yes	6 (33.3)	25 (27.5)	1.42 (0.47-4.24)	0.532
Altered consciousness[a]				
No	1 (5.6)	25 (27.5)	1.00	
Yes	17 (94.4)	66 (72.5)	6.44 (0.81-50.96)	0.078
Seizures				
No	7 (38.9)	40 (44.0)	1.00	
Yes	11 (61.1)	51 (56.0)	1.23 (0.44-3.47)	0.692
Shortness of breath				
No	15 (83.3)	79 (86.8)	1.00	
Yes	2 (11.1)	12 (13.2)	0.88 (0.18-4.33)	0.873
Persistent cough				
No	13 (72.2)	54 (59.3)	1.00	
Yes	5 (27.8)	37 (40.7)	0.56 (0.18-1.71)	0.309
Poor weight gain / weight loss				
No	7 (38.9)	49 (53.8)	1.00	
Yes	10 (55.6)	41 (45.1)	1.71 (0.60-4.88)	0.319
Duration of symptoms				
0-7 days	7 (38.9)	30 (33.0)	1.00	
8-14 days	8 (44.4)	50 (54.9)	0.69 (0.23-2.08)	0.506
>14 days	3 (16.7)	7 (7.7)	1.84 (0.38-8.94)	0.452

Patient characteristics	Dead (n=18)	Alive (n=91)	cOR (95% CI)	p-value
Examination findings at baseline				
Body temperature				
<38 °C	12 (66.7)	76 (83.5)	1.00	
≥38 °C	6 (33.3)	15 (16.5)	2.53 (0.82-7.81)	0.106
Respiration rate				
<25/min	6 (33.3)	36 (39.6)	1.36 (0.47-3.95)	0.574
≥25/min	12 (66.7)	53 (58.2)	1.00	
Involuntary movement				
No	16 (88.9)	80 (87.9)	1.00	
Yes	1 (5.6)	8 (8.8)	0.62 (0.07-5.35)	0.668
Cranial nerve palsies				
No	16 (88.9)	73 (80.2)	1.00	
Yes	1 (5.6)	16 (17.6)	0.28 (0.03-2.31)	0.240
Any type of motor deficit				
No	5 (27.8)	35 (38.5)	1.00	
Yes	11 (61.1)	47 (51.6)	1.64 (0.52-5.14)	0.398
Unequal pupils				
No	16 (88.9)	88 (96.7)	1.00	
Yes	1 (5.6)	1 (1.1)	5.50 (0.33-92.51)	0.237
Signs of upper motor neuron lesion				
No	3 (16.7)	15 (16.5)	1.00	
Yes	13 (72.2)	68 (74.7)	0.96 (0.24-3.78)	0.949
Signs of raised intracranial pressure[a,c]				
No	12 (66.7)	83 (91.2)	1.00	
Yes	6 (33.3)	8 (8.8)	5.19 (1.53-17.56)	0.008
CSF findings				
Leucocyte ≥10 cells/μL				
No	3 (16.7)	21 (23.1)	1.00	
Yes	15 (83.3)	67 (73.6)	1.57 (0.41-5.94)	0.509
Leucocyte ≥100 cells/μL				
No	9 (50.0)	61 (67.0)	1.00	
Yes	9 (50.0)	27 (29.7)	2.26 (0.81-6.32)	0.121
Lymphocytic predominance >50%				
No	3 (16.7)	18 (19.8)	1.00	
Yes	15 (83.3)	69 (75.8)	1.30 (0.34-5.00)	0.698
Protein >100 mg/dL				
No	7 (38.9)	39 (42.9)	1.00	
Yes	11 (61.1)	49 (53.8)	1.25 (0.44-3.52)	0.672
Glucose <40 mg/dL				
No	10 (55.6)	54 (59.3)	1.00	
Yes	7 (38.9)	29 (31.9)	1.30 (0.45-3.78)	0.626
CSF/blood glucose ratio <50%				
No	7 (38.9)	33 (36.3)	1.00	
Yes	7 (38.9)	35 (38.5)	0.94 (0.30-2.98)	0.920
Radiological findings				
Chest radiography				
Normal	5 (27.8)	38 (41.8)	1.00	
Miliary TB	1 (5.6)	6 (6.6)	1.27 (0.12-12.80)	0.841
Other signs of TB	12 (66.7)	47 (51.6)	1.94 (0.63-5.99)	0.249
Hydrocephalus[a,c]				
No	3 (16.7)	66 (72.5)	1.00	
Yes	13 (72.2)	23 (25.3)	12.43 (3.25-47.59)	<0.001
Neurosurgery in hydrocephalus patients[§§]				
No	11 (84.6)	10 (43.5)	7.15 (1.28-39.83)	0.025
Yes	2 (15.4)	13 (56.5)	1.00	
Basal meningeal enhancement				
No	6 (33.3)	42 (46.2)	1.00	
Yes	10 (55.6)	47 (51.6)	1.49 (0.50-4.45)	0.476
Cerebral infarct				
No	14 (77.8)	84 (92.3)	1.00	
Yes	2 (11.1)	5 (5.5)	2.40 (0.42-13.60)	0.323
Tuberculoma[a]				
No	12 (66.7)	85 (93.4)	1.00	
Yes	4 (22.2)	4 (4.4)	7.08 (1.56-32.13)	0.011
At least 1 sign found on CT scan[a]				
No	1 (5.6)	29 (31.9)	1.00	
Yes	15 (83.3)	60 (65.9)	7.25 (0.91-57.58)	0.061
Bacteriological findings				
TST positive[a]				

Patient characteristics	Dead (n=18)	Alive (n=91)	cOR (95% CI)	p-value
No	10 (55.6)	76 (83.5)	1.00	
Yes	8 (44.4)	15 (16.5)	4.05 (1.37-11.96)	0.011
GeneXpert MTB/RIF testing				
Negative	11 (61.1)	60 (65.9)	1.00	
M.tb identified from CSF	5 (27.8)	19 (20.9)	1.43 (0.44-4.65)	0.547
M.tb identified from non-CSF	2 (11.1)	6 (6.6)	1.82 (0.32-10.20)	0.497
AFB smear microscopy				
Negative	15 (83.3)	74 (81.3)	1.00	
Positive from CSF	2 (11.1)	12 (13.2)	0.82 (0.17-4.06)	0.810
Positive from non-CSF	1 (5.6)	5 (5.5)	0.99 (0.11-9.06)	0.991
M. tb cultured from any source				
No	14 (77.8)	79 (86.8)	1.00	
Yes	4 (22.2)	10 (11.0)	2.26 (0.62-8.21)	0.217
In-hospital complications				
Motor disorders[a]				
No	7 (38.9)	60 (65.9)	1.00	
Yes	11 61.1)	31 (34.1)	3.04 (1.07-8.62)	0.036
Visual impairment				
No	18 (100.0)	85 (93.4)	1.00	
Yes	0 (0.0)	6 (6.6)	n/a	0.999
Hearing impairment				
No	18 (100.0)	87 (95.6)	1.00	
Yes	0 (0.0)	4 (4.4)	n/a	0.999
Neurodevelopmental delay				
No	16 (88.9)	80 (87.9)	1.00	
Yes	2 (11.1)	11 (12.1)	0.91 (0.18-4.50)	0.907
Epileptic seizures				
No	17 (94.4)	82 (90.1)	1.00	
Yes	1 (5.6)	9 (9.9)	0.54 (0.06-4.51)	0.566
Anti-TB drug-induced hepatotoxicity				
No	14 (77.8)	76 (83.5)	1.00	
Yes	4 (22.2)	15 (16.5)	1.45 (0.42-5.01)	0.559
Others				
Oral corticosteroid				
No	0 (0.0)	4 (4.4)	n/a	0.999
Yes	17 (94.4)	85 (93.4)	1.00	
Physiotherapy				
No	14 (77.8)	62 (68.1)	1.73 (0.45-6.58)	0.421
Yes	3 (16.7)	23 (25.3)	1.00	

cOR: crude odds ratio, AFB: acid-fast bacilli, BCG: Bacillus Calmette-Guerin, CSF: cerebrospinal fluid, CI: confidence interval, GCS: Glasgow Coma Scale, IQR: interquartile rage, TB: tuberculosis, TBM: tuberculous meningitis, TST: tuberculin skin test.

[§]In children aged <5 years, moderate malnutrition was defined as weight-for-age or height-for-age Z-scores ≥-3 and <-2 standard deviations (SD), and severe malnutrition as weight-for-age or height-for-age Z-scores <-3 SD. In children aged 5-14 years, moderate malnutrition was defined as height-for-age or BMI-for-age Z-scores ≥-3 and <-2 SD, and severe malnutrition as height-for-age or BMI-for-age Z-scores <-3 SD (*5*).

[¥]Diagnostic score was assessed using a uniform case definition criteria for TBM, and was categorized as definite TBM (microbiologically proven from CSF examination), probable TBM (diagnostic score of ≥10 points when cerebral imaging is not available or ≥12 points when cerebral imaging is available), and possible TBM (diagnostic score of 6-9 points when cerebral imaging is not available or 6-11 points when cerebral imaging is available) (*2*).

[¶]Severity of TBM was classified according to the modified British Medical Research Council grading system as stage I (GCS of 15 with no focal neurologic signs), stage II (GCS of 11-14 or 15 with focal neurologic signs), or stage III (GCS ≤10) (*7*).

[§§]Analysis was only performed in patients with hydrocephalus

[a]Variables eligible for inclusion in multivariate analysis.

[b,c]Due to the likelihood of collinearity (TBM stage *vs.* GCS score and hydrocephalus *vs.* signs of raised intracranial pressure), only one of each of these variables was included during the development of the final multivariate model.

Appendix Table 6. Univariate logistic regression model for predictors of severe neurologic sequelae at tuberculous meningitis treatment completion in children treated for tuberculous meningitis at Hasan Sadikin Hospital, Bandung, Indonesia, 2011–2020

	Severe neurologic sequelae			
Patient characteristics	Yes (n=33)	No (n=58)	cOR (95% CI)	p-value
Year of diagnosis (median (IQR))	2018 (2016-2018)	2018 (2016-2019)	1.05 (0.86-1.28)	0.628
Year of diagnosis				
2011-2015	3 (9.1)	11 (19.0)	0.43 (0.11-1.66)	0.219
2016-2020	30 (90.9)	47 (81.0)	1.00	
Age, years (median (IQR))			0.93 (0.85-1.02)	0.138
Age[a]				
<2 years	13 (39.4)	13 (22.4)	2.78 (0.94-8.20)	0.064
2-4 years	2 (6.1)	7 (12.1)	0.79 (0.14-4.55)	0.795
5-9 years	9 (27.3)	13 (22.4)	1.92 (0.61-6.02)	0.261
10-14 years	9 (27.3)	25 (43.1)	1.00	

Patient characteristics	Severe neurologic sequelae		cOR (95% CI)	p-value
	Yes (n=33)	No (n=58)		
Sex[a]				
Male	12 (36.4)	27 (46.6)	0.66 (0.27-1.58)	0.346
Female	21 (63.6)	31 (53.4)	1.00	
Parent's last education				
Junior high school or lower	17 (51.5)	23 (39.7)	1.62 (0.68-3.82)	0.275
Senior high school or higher	16 (48.5)	35 (60.3)	1.00	
Parent's monthly income				
USD ≤140,00	17 (51.5)	35 (60.3)	0.69 (0.28-1.70)	0.424
USD >140,00	14 (42.4)	20 (34.5)	1.00	
Area of living				
Urban	12 (36.4)	28 (48.3)	1.00	
Rural	20 (60.6)	29 (50.0)	1.61 (0.66-3.90)	0.292
Weight-for-age Z-score				
≥-2 (normal)	13 (39.4)	21 (36.2)	1.00	
<-2 (underweight)	16 (48.5)	21 (36.2)	1.23 (0.48-3.18)	0.668
Height-for-age Z-score				
≥-2 (normal)	19 (57.6)	34 (58.6)	1.00	
<-2 (stunted)	14 (42.4)	24 (41.4)	1.04 (0.44-2.48)	0.923
Weight-for-height Z-score				
≥-2 (normal)	14 (42.4)	33 (56.9)	1.00	
<-2 (wasted)	19 (57.6)	25 (43.1)	1.79 (0.75-4.25)	0.186
BMI-for-age Z-score				
≥-2 (normal)	14 (42.4)	33 (56.9)	1.00	
<-2 (low BMI)	19 (57.6)	25 (43.1)	1.79 (0.75-4.25)	0.186
Nutritional status[§]				
Normal	9 (27.3)	21 (36.2)	1.00	
Moderate malnutrition	12 (36.4)	17 (29.3)	1.65 (0.56-4.83)	0.363
Severe malnutrition	12 (36.4)	20 (34.5)	1.40 (0.48-4.04)	0.534
Known BCG vaccination				
No	3 (9.1)	12 (20.7)	0.38 (0.10-1.47)	0.163
Yes	30 (90.9)	46 (79.3)	1.00	
Known TB contact history				
No	25 (75.8)	43 (74.1)	1.00	
Yes	8 (24.2)	15 (25.9)	0.92 (0.34-2.47)	0.864
TBM category and stage				
TBM category[¥]				
Definite TBM	6 (18.2)	13 (22.4)	1.00	
Probable TBM	22 (66.7)	32 (55.2)	1.49 (0.49-4.52)	0.481
Possible TBM	5 (15.2)	13 (22.4)	0.83 (0.20-3.43)	0.800
TBM stage[¶,a]				
Stage I	5 (15.2)	17 (29.3)	1.00	
Stage II	14 (42.4)	31 (53.4)	1.53 (0.47-5.00)	0.476
Stage III	14 (42.4)	10 (17.2)	4.76 (1.32-17.22)	0.017
GCS (median (IQR))			0.91 (0.76-1.09)	0.316
Presenting symptoms				
Fever				
No	2 (6.1)	5 (8.6)	1.00	
Yes	31 (93.9)	53 (91.4)	1.46 (0.27-7.99)	0.661
Severe headache				
No	23 (69.7)	36 (62.1)	1.00	
Yes	9 (27.3)	21 (36.2)	0.67 (0.26-1.72)	0.405
Muscle weakness				
No	23 (69.7)	42 (72.4)	1.00	
Yes	9 (27.3)	16 (27.6)	1.03 (0.39-2.69)	0.956
Altered consciousness				
No	9 (27.3)	16 (27.6)	1.00	
Yes	24 (72.7)	42 (72.4)	1.02 (0.39-2.65)	0.974
Seizures				
No	13 (39.4)	27 (46.6)	1.00	
Yes	20 (60.6)	31 (53.4)	1.34 (0.56-3.19)	0.509
Shortness of breath				
No	30 (90.9)	49 (84.5)	1.00	
Yes	3 (9.1)	9 (15.5)	0.54 (0.14-2.17)	0.389
Persistent cough				
No	21 (63.6)	33 (56.9)	1.00	
Yes	12 (36.4)	25 (43.1)	0.75 (0.31-1.82)	0.530
Poor weight gain / weight loss				
No	14 (42.4)	35 (60.3)	1.00	

Patient characteristics	Severe neurologic sequelae Yes (n=33)	No (n=58)	cOR (95% CI)	p-value
Yes	18 (54.5)	23 (39.7)	1.96 (0.82-4.69)	0.132
Duration of symptoms				
0-7 days	14 (42.4)	16 (27.6)	1.00	
8-14 days	16 (48.5)	34 (58.6)	0.54 (0.21-1.36)	0.192
>14 days	3 (9.1)	4 (6.9)	0.86 (0.16-4.51)	0.856
Examination findings at baseline				
Body temperature				
<38 °C	23 (69.7)	53 (91.4)	1.00	
≥38 °C	10 (30.3)	5 (8.6)	4.61 (1.42-14.99)	0.011
Respiration rate				
<25/min	12 (36.4)	24 (41.4)	1.00	
≥25/min	21 (63.6)	32 (55.2)	1.31 (0.54-3.18)	0.547
Involuntary movement				
No	29 (87.9)	51 (87.9)	1.00	
Yes	4 (12.1)	4 (6.9)	1.76 (0.41-7.56)	0.448
Cranial nerve palsies				
No	26 (78.8)	47 (81.0)	1.00	
Yes	7 (21.2)	9 (15.5)	1.41 (0.47-4.21)	0.543
Any type of motor deficit[a]				
No	8 (24.2)	27 (46.6)	1.00	
Yes	24 (72.7)	23 (39.7)	3.52 (1.33-9.33)	0.011
Unequal pupils				
No	32 (97.0)	56 (96.6)	1.00	
Yes	1 (3.0)	0 (0.0)	n/a	1.000
Signs of upper motor neuron lesions				
No	5 (15.2)	10 (17.2)	1.00	
Yes	27 (81.8)	41 (70.7)	1.32 (0.40-4.28)	0.647
Signs of raised intracranial pressure				
No	31 (93.9)	52 (89.7)	1.00	
Yes	2 (6.1)	6 (10.3)	0.56 (0.11-2.94)	0.493
CSF findings				
Leucocyte ≥10 cells/µL				
No	9 (27.3)	12 (20.7)	1.00	
Yes	23 (69.7)	44 (75.9)	0.70 (0.26-1.90	0.479
Leucocyte ≥100 cells/µL				
No	24 (72.7)	37 (63.8)	1.00	
Yes	8 (24.2)	19 (32.8)	0.65 (0.24-1.72)	0.384
Lymphocytic predominance >50%				
No	9 (27.3)	9 (15.5)	1.00	
Yes	23 (69.7)	46 (79.3)	0.50 (0.17-1.43)	0.196
Protein >100 mg/dL				
No	11 (33.3)	28 (48.3)	1.00	
Yes	21 (63.6)	28 (48.3)	1.91 (0.78-4.69)	0.158
Glucose <40 mg/dL				
No	20 (60.6)	34 (58.6)	1.00	
Yes	12 (36.4)	17 (29.3)	1.20 (0.48-3.02)	0.699
CSF/blood glucose ratio <50%				
No	13 (39.4)	20 (34.5)	1.00	
Yes	15 (45.5)	20 (34.5)	1.15 (0.44-3.04)	0.772
Radiological findings				
Chest radiography[a]				
Normal	9 (27.3)	29 (50.0)	1.00	
Miliary TB	3 (9.1)	3 (5.2)	3.22 (0.55-18.85)	0.194
Other signs of TB	21 (63.6)	26 (44.8)	2.60 (1.01-6.68)	0.047
Hydrocephalus[a]				
No	22 (66.7)	44 (75.9)	1.00	
Yes	11 (33.3)	12 (20.7)	1.83 (0.70-4.81)	0.218
Neurosurgery in hydrocephalus patients[§§]				
No	4 (36.4)	6 (50.0)	0.57 (0.11-3.04)	0.511
Yes	7 (63.6)	6 (50.0)	1.00	
Basal meningeal enhancement				
No	14 (42.4)	28 (48.3)	1.00	
Yes	19 (57.6)	28 (48.3)	1.36 (0.57-3.23)	0.490
Cerebral infarct				
No	30 (90.9)	54 (93.1)	1.00	
Yes	3 (9.1)	2 (3.4)	2.70 (0.43-17.07)	0.291
Tuberculoma				
No	31 (93.9)	54 (93.1)	1.00	

Patient characteristics	Severe neurologic sequelae Yes (n=33)	No (n=58)	cOR (95% CI)	p-value
Yes	2 (6.1)	2 (3.4)	1.74 (0.23-12.99)	0.588
At least 1 sign found on CT scan[a]				
No	7 (21.2)	22 (37.9)	1.00	
Yes	26 (78.8)	34 (58.6)	2.40 (0.89-6.48)	0.083
Bacteriological findings				
TST positive				
No	26 (78.8)	50 (86.2)	1.00	
Yes	7 (21.2)	8 (13.8)	1.68 (0.55-5.15)	0.362
GeneXpert MTB/RIF testing				
Negative	23 (69.7)	37 (63.8)	1.00	
M.tb identified from CSF	6 (18.2)	13 (22.4)	0.74 (0.25-2.23)	0.595
M.tb identified from non-CSF	4 (12.1)	2 (3.4)	3.22 (0.54-18.99)	0.197
AFB smear microscopy[a]				
Negative	23 (69.7)	51 (87.9)	1.00	
Positive from non-CSF	8 (24.2)	4 (6.9)	4.43 (1.21-16.23)	0.024
M. tb cultured from any source				
No	28 (84.8)	51 (87.9)	1.00	
Yes	5 (15.2)	5 (8.6)	1.82 (0.48-6.84)	0.374
Others				
Anti-TB drug-induced hepatotoxicity				
No	29 (87.9)	47 (81.0)	1.00	
Yes	4 (12.1)	11 (19.0)	0.59 (0.17-2.02)	0.401
Oral corticosteroid				
No	0 (0.0)	4 (6.9)	n/a	0.999
Yes	33 (100.0)	52 (89.7)	1.00	
Physiotherapy				
No	21 (63.6)	41 (70.7)	0.56 (0.21-1.48)	0.241
Yes	11 (33.3)	12 (20.7)	1.00	

cOR: crude odds ratio, AFB: acid-fast bacilli, BCG: Bacillus Calmette-Guerin, CSF: cerebrospinal fluid, CI: confidence interval, GCS: Glasgow Coma Scale, IQR: interquartile rage, TB: tuberculosis, TBM: tuberculous meningitis, TST: tuberculin skin test.
[§]In children aged <5 years, moderate malnutrition was defined as weight-for-age or height-for-age Z-scores ≥-3 and <-2 standard deviations (SD), and severe malnutrition as weight-for-age or height-for-age Z-scores <-3 SD. In children aged 5-14 years, moderate malnutrition was defined as height-for-age or BMI-for-age Z-scores ≥-3 and <-2 SD, and severe malnutrition as height-for-age or BMI-for-age Z-scores <-3 SD (5).
[¥]Diagnostic score was categorized as definite TBM (microbiologically proven from CSF examination), probable TBM (diagnostic score of ≥10 points when cerebral imaging is not available or ≥12 points when cerebral imaging is available), and possible TBM (diagnostic score of 6-9 points when cerebral imaging is not available or 6-11 points when cerebral imaging is available) (*2*).
[¶]Severity of TBM was classified as stage I (GCS of 15 with no focal neurologic signs), stage II (GCS of 11-14 or 15 with focal neurologic signs), or stage III (GCS ≤10) (7).
[§§]Analysis was only performed in patients with hydrocephalus
[a]Variables eligible for inclusion in multivariate analysis.

References

1. Nataprawira HM, Ruslianti V, Solek P, Hawani D, Milanti M, Anggraeni R, et al. Outcome of tuberculous meningitis in children: the first comprehensive retrospective cohort study in Indonesia. Int J Tuberc Lung Dis. 2016;20:909–14. PubMed https://doi.org/10.5588/ijtld.15.0555

2. Marais S, Thwaites G, Schoeman JF, Török ME, Misra UK, Prasad K, et al. Tuberculous meningitis: a uniform case definition for use in clinical research. Lancet Infect Dis. 2010;10:803–12. PubMed https://doi.org/10.1016/S1473-3099(10)70138-9

3. World Health Organization. Global tuberculosis report 2020. 2020 [cited 2021 Dec 28]. https://www.who.int/publications/i/item/9789240013131

4. Marais BJ, Gie RP, Schaaf HS, Hesseling AC, Obihara CC, Starke JJ, et al. The natural history of childhood intrathoracic tuberculosis—a critical review of the pre-chemotherapy literature. Int J Tuberc Lung Dis. 2004;8:392–402. PubMed

5. de Onis M, Onyango AW, Borghi E, Siyam A, Nishida C, Siekmann J. Development of a WHO growth reference for school-aged children and adolescents. Bull World Health Organ. 2007;85:660–7. PubMed https://doi.org/10.2471/BLT.07.043497

6. World Health Organization. WHO child growth standards: length/height-for-age, weight-for-age, weight-for-length, weight- for-height and body mass index-for-age: methods and development. 2006 [cited 2021 Dec 28]. https://www.who.int/publications/i/item/924154693X

7. Thwaites GE, Nguyen DB, Nguyen HD, Hoang TQ, Do TTO, Nguyen TCT, et al. Dexamethasone for the treatment of tuberculous meningitis in adolescents and adults. N Engl J Med. 2004;351:1741–51. PubMed https://doi.org/10.1056/NEJMoa040573

8. Faried A, Arief G, Arifin MZ, Nataprawira HM. Correlation of lactate concentration in peripheral plasma and cerebrospinal fluid with Glasgow outcome scale for patients with tuberculous meningitis complicated by acute hydrocephalus treated with fluid diversions. World Neurosurg. 2018;111:e178–82. PubMed https://doi.org/10.1016/j.wneu.2017.12.007

9. Saukkonen JJ, Cohn DL, Jasmer RM, Schenker S, Jereb JA, Nolan CM, et al.; ATS (American Thoracic Society) Hepatotoxicity of Antituberculosis Therapy Subcommittee. An official ATS statement: hepatotoxicity of antituberculosis therapy. Am J Respir Crit Care Med. 2006;174:935–52. PubMed https://doi.org/10.1164/rccm.200510-1666ST

10. Garna H, Nataprawira HM. Guidelines for diagnosis and therapy in pediatrics, 6th edition [Pedoman diagnosis dan terapi ilmu kesehatan anak, edisi ke-6]. Bandung (Indonesia): Universitas Padjadjaan/Hasan Sadikin Hospital; 2014. p. 1169–87.
